# Aged murine bone marrow myeloid and mesenchymal cells develop unique senescence phenotypes

**DOI:** 10.1172/JCI195772

**Published:** 2026-01-27

**Authors:** Madison L. Doolittle, Mitchell N. Froemming, Jennifer L. Rowsey, Ming Ruan, Leena Sapra, Joshua N. Farr, David G. Monroe, Sundeep Khosla

**Affiliations:** 1 Division of Endocrinology and; 2 Robert and Arlene Kogod Center on Aging, Mayo Clinic, Rochester, Minnesota, USA.; 3 Center for Regenerative Medicine and Skeletal Development, UConn Health, Farmington, Connecticut, USA.; 4 Department of Physiology and Biomedical Engineering, Mayo Clinic, Rochester, Minnesota, USA.; 5 Department of Medicine, University of Arizona, Tucson, Arizona, USA.

**Keywords:** Aging, Bone biology, Cellular senescence

## Abstract

Cellular senescence is a heterogeneous phenotype characterized primarily in mesenchymal cells, but the extent to which immune cells differ in their senescence phenotype, or “senotype,” is unclear. Here, we applied single-cell approaches alongside both global and cell-specific genetic senolytic mouse models to evaluate the senotype of immune cells in the bone marrow of aging mice. We found that myeloid-lineage cells exhibited the highest expression of p16 and senescence-associated secretory phenotype markers among all immune cell types. In contrast with clearance of p16^+^ senescent mesenchymal cells, targeted clearance of p16^+^ myeloid cells in aged mice had only minor effects on age-related bone loss in male mice, with no effects in females. In more detailed analyses, p16^+^ myeloid cells were only acutely cleared, being repopulated back to basal levels within a short time. This led to a lack of long-lasting reduction in senescent cell burden, unlike when targeting bone mesenchymal cells. In vitro, myeloid-lineage cells differed markedly from mesenchymal cells in the development of a senescent phenotype. Collectively, our findings indicate that aged bone marrow myeloid cells do not achieve the fully developed senescent phenotype originally described in mesenchymal cells, justifying further characterization of senotypes of immune cells across tissues.

## Abstract 2

Celllular senescence causes age-related tissue damage, but this process has largely been characterized in mesenchymal cells. In this study, we compare the senescence phenotype of murine bone/bone marrow mesenchymal versus immune cells and find important differences between them. This has important implications for our understanding of cellular senescence across tissues as well as clinical implications for strategies to clear these cells and improve age-related pathologies.

## Graphical abstract

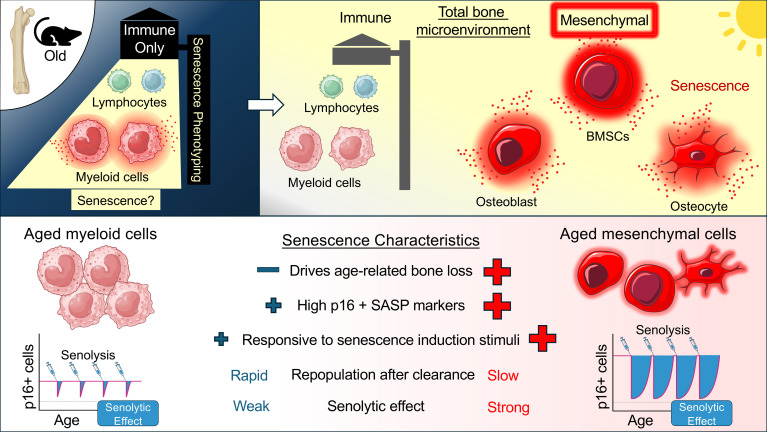

## Introduction

Cellular senescence is classically defined as a state of growth arrest resulting from proliferative exhaustion (1), later found to be accompanied by a senescence-associated secretory phenotype (SASP) that drives tissue dysfunction and disease (2, 3). The accumulation of senescent cells within various tissues is associated with advancing biological age (4, 5), and clearance of these cells in old mice ameliorates age-related diseases (6–8). Recent findings indicate that cells can differ greatly in their senescence phenotype, or “senotype,” with functions resulting in detrimental (9–11), beneficial (12–14), or null (15, 16) effects on tissue homeostasis. Thus, to guide future senolytic therapies, recent efforts have largely focused on identifying which cells in each tissue microenvironment become senescent with age and the subsequent characterization of their respective senotypes (17–19).

Cellular senescence was originally described in mesenchymal cells (20), but over time other cell types, including endothelial (21) as well as immune cells (22), were found to express features of senescence. The onset of cellular senescence within immune cells, however, has been a topic of debate (23–26). This is largely due to the challenges in distinguishing cellular senescence from “inflammaging,” as inflammatory factors released by aged immune cells (e.g., interleukins, cytokines, chemokines) (27–29) greatly overlap with SASP proteins (30), and certain immune subtypes, particularly macrophages, can express senescence markers in a reversible manner, dependent on their activation state (31). Terminology is an additional issue, as “immunosenescence” refers to the functional decline in immune capacity observed with age, regardless of whether the afflicted cell type is in a true state of cellular senescence (23, 32). It is also unclear whether immune cell types, whose lifespans are often less than a week (33), have the capacity to persist and perpetuate disease in a state of cellular senescence similar to longer lived mesenchymal cells (34). Although the induction of DNA damage in immune cells does induce widespread senescence in mesenchymal cells (35), it remains unclear whether these immune cells themselves are senescent, and if so, the extent to which this influences diseases of aging.

A previous study by Sladitschek-Martens et al. (26) provided an important clue that senescence in immune cells may differ fundamentally from that found in mesenchymal cells. Specifically, these investigators linked the senescence phenotype with aging to age-related reductions in YAP/TAZ signaling, which then led to loss of both lamin B1 and the nuclear envelope as well as upregulation of the SASP. Importantly, these investigators noted that the age-related reduction in YAP/TAZ signaling was limited to mesenchymal cells and not present in immune cells, but they did not pursue the potential senescence phenotype of immune cells in their work. As such, there is currently a fundamental gap in our understanding of how senescence in immune cells may be similar to or different from that in mesenchymal cells, where it was originally described (1). Thus, in this study, we performed comprehensive phenotyping of cellular senescence in murine bone marrow immune cells to determine the extent to which these cells exhibited senescence akin to mesenchymal cells. To distinguish from senescence-independent inflammation, we applied multiparametric senescence phenotyping at the single-cell level to identify major criteria for cellular senescence (e.g., growth arrest, apoptosis resistance, SASP) within the same cells. Additionally, we leveraged genetic cell-specific senolytic mouse models to determine functional contributions of senescent cell subtypes toward tissue inflammation and aging phenotypes. We then compared key differences between immune and mesenchymal cells expressing senescence markers within the same tissue (bone/bone marrow), uncovering phenotypes that may explain their relative contributions to biological aging.

## Results

### Myeloid cells express the highest levels of senescence markers in aged murine bone marrow.

**Figure 1:**
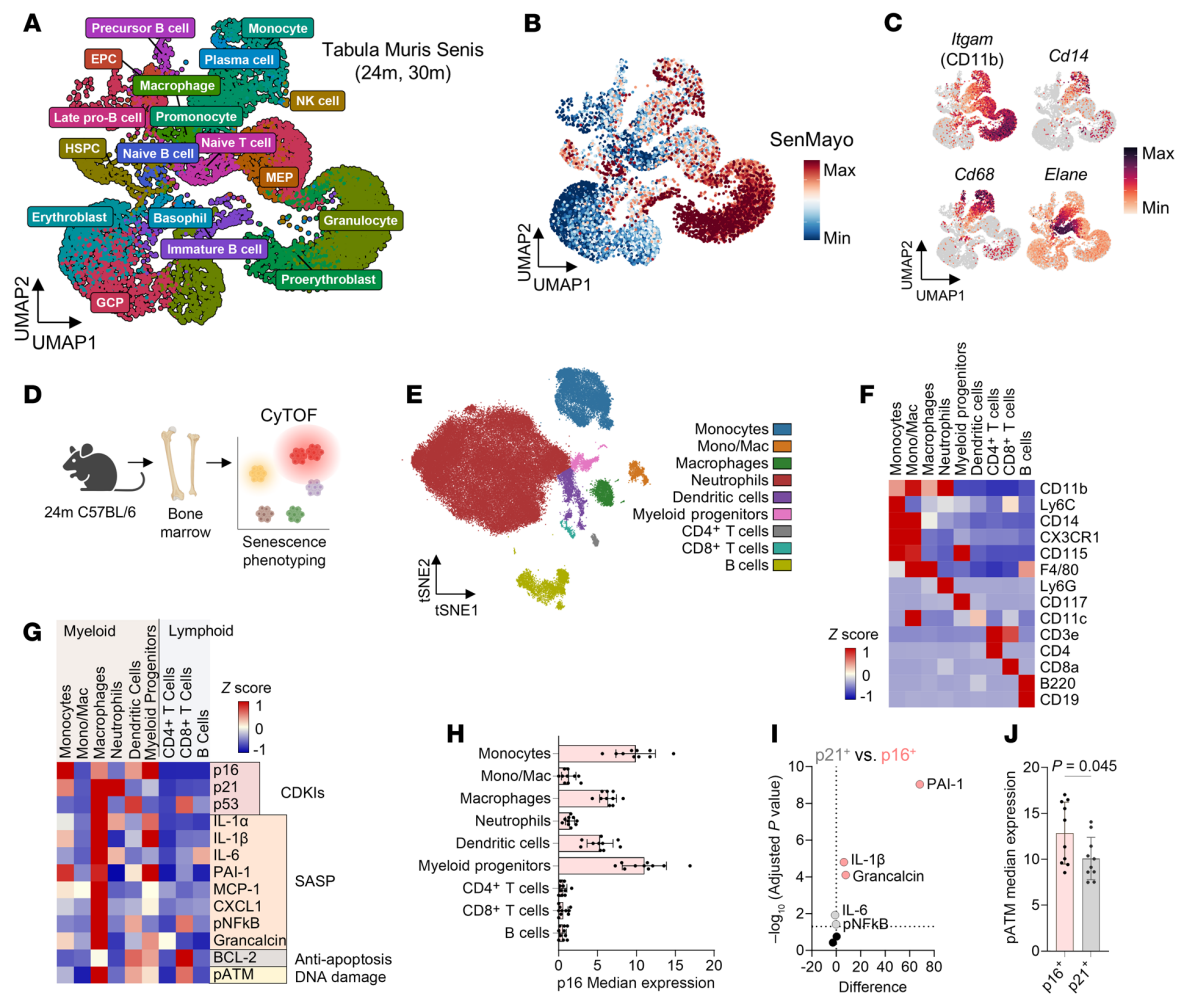
Myeloid-lineage cells express senescence markers in aged murine bone marrow. (**A**) Uniform manifold approximation and projection (UMAP) visualization of clustered bone marrow cell scRNA-seq data from the Tabula Muris Senis database, displaying only samples from 24-month (24m) and 30-month (30m) mouse cohorts. *N* = 4 male mice per age group. (**B**) SenMayo gene enrichment score and (**C**) individual myeloid gene expression plots overlaid on UMAP plots. (**D**) Schematic for senescence phenotyping of freshly isolated aged murine bone marrow cells by CyTOF. *N* = 10 female 24-month-old mice. (**E**) t-Distributed stochastic neighbor embedding (t-SNE) visualization of clustered bone marrow cells analyzed by CyTOF. (**F** and **G**) Heatmaps displaying median expression of (**F**) identity markers and (**G**) senescence markers across immune clusters. (**H**) p16 median expression across immune clusters. (**I**) Volcano plot of SASP marker expression differences between p16^+^ and p21^+^ immune cells. Significance was determined by multiple *t* tests with Holm-Šídák correction. (**J**) pATM median expression between p16^+^ and p21^+^ immune cells. Significance was determined by unpaired *t* test.

To determine the bone marrow immune cell types exhibiting senescence markers with aging, we first explored single-cell RNA-sequencing (scRNA-seq) datasets of bone marrow from aged mice (24–30 months old) generated by the Tabula Muris Consortium (36) (Figure 1A). Gene enrichment analysis of the SenMayo gene set (30) identified several clusters highly expressing senescence markers (Figure 1B), with the strongest signal within myeloid-lineage cells (*Itgam* [CD11b]^+^) (Figure 1C), consistent with previous findings in human bone marrow (30). Notably, we found strong overlap between expression of *Cdkn2a* (encoding p16) and the SenMayo gene set within *Cd14*^+^ monocytes/macrophages (Supplemental Figure 1, A and B; supplemental material available online with this article; https://doi.org/10.1172/JCI195772DS1). Expression of other senescence-related genes *Cdkn1a* (p21) and *Trp53* (p53) did not appear to overlap with SenMayo enrichment or *Cdkn2a* expression (Supplemental Figure 1C). *Cdkn2a* was expressed predominantly in myeloid-lineage cells (Supplemental Figure 1, A–C), and *Cdkn2a^+^* cells increased in proportion with age (Supplemental Figure 1D). In addition, using the weighted single-cell transcriptomic senescence scoring system *Senepy* (37), we also found strong enrichment in SenePy genes in myeloid-lineage cells, particularly within macrophage, pro-monocyte, and monocyte clusters (Supplemental Figure 1, E and F).

To investigate this further, we next performed senescence phenotyping using mass cytometry (CyTOF) on bone marrow isolated from old (24-month-old) wild-type (WT) mice (Figure 1D; see Supplemental Figure 2A for the CyTOF gating strategy). We previously validated a panel of antibodies for CyTOF (17), including the mouse p16 antibody. Specifically, we demonstrated that this antibody gave an excellent signal-to-noise ratio by CyTOF when mouse p16 was expressed in human U2Os cells (17). In addition, our gating strategy used cells from p16-knockout mice (38) as a negative control. Finally, this particular p16 antibody has now been extensively used by multiple other established laboratories in the senescence field (11, 39, 40). Thus, alongside our previously validated senescence antibodies (17), we applied an immune panel that identified key immune populations in the bone marrow (Figure 1, E and F). Among these populations, the majority of senescence-associated proteins were strongly expressed by myeloid-lineage cells, specifically monocytes (CD11b^+^, Ly6C^+^, CD14^+^), macrophages (CD11b^+^, F4/80^+^), and myeloid progenitors (CD117^+^, CD115^+^) (Figure 1G). Interestingly, these cells also demonstrated high levels of p16 (Figure 1, G and H), a senescence-associated protein expressed by cells that functionally contribute to age-related bone loss (41, 42). Although certain immune cells also expressed p21 (Supplemental Figure 2B), we found that p16^+^ immune cells demonstrated higher levels of SASP proteins (Figure 1I and Supplemental Figure 2C) and the DNA damage marker pATM (Figure 1J) than p21^+^ cells. Additionally, we recently found that global clearance of p21^+^ cells fails to prevent age-related bone loss (16), suggesting that the role of p21 in age-related skeletal senescence may be minor. Other senescence markers demonstrated strong coexpression within p16^+^ myeloid-lineage clusters (Supplemental Figure 2D). Thus, among possible immune cell types within the bone microenvironment, we hypothesized that p16^+^ myeloid-lineage cells had the highest likelihood of being the candidate senescent population influencing skeletal aging.

### Clearance of p16^+^ myeloid-lineage cells has limited effects on skeletal aging.

**Figure 2:**
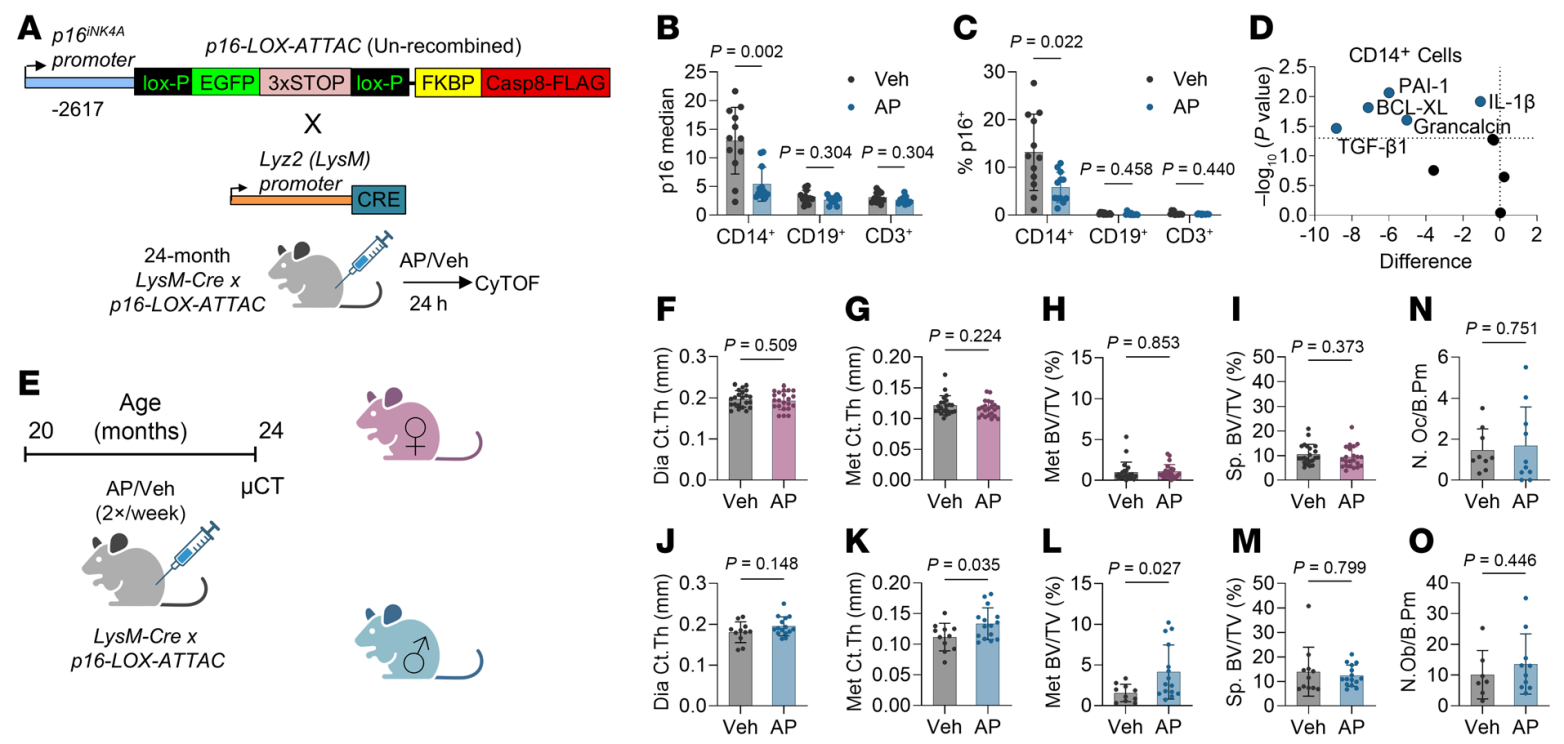
Clearance of p16^+^ myeloid cells has limited effects on age-related bone loss in male mice with no effects in females. (**A**) Schematic of the un-recombined p16-LOX-ATTAC transgene crossed with the Lyz2 (LysM)-Cre. These LysM-LOX-ATTAC mice were then aged to 24 months, and bone marrow was harvested for CyTOF analysis at 24 hours after treatment with either vehicle or AP. *n* = 12 mice per treatment (6 male, 6 female). (**B**) p16 median expression and (**C**) %p16^+^ cells quantified in myeloid cells (CD14^+^), B cells (CD19^+^), and T cells (CD3^+^). (**D**) Volcano plot of differences in senescence-associated factor expression in CD14^+^ cells in AP- versus vehicle-treated mice. (**E**) Schematic of LysM-LOX-ATTAC bone phenotyping cohort, which were treated with vehicle or AP twice weekly from 20 to 24 months old. *N* = 44 females (*N* = 22 vehicle, *N* = 22 AP). *N* = 26 males (*N* = 11 vehicle, *N* = 15 AP). (**F**–**M**) μCT analyses in female (**F**–**I**) and male (**J**–**M**) mice. Cortical thickness (Ct.Th) at the diaphysis (**F** and **J**) and metaphysis (**G** and **K**) of the femur, as well as trabecular bone volume over total volume (BV/TV) at the femur metaphysis (**H** and **L**) and lumbar spine (**I** and **M**). (**N**) Number of osteoclasts per bone perimeter (N.Oc/B.Pm) or (**O**) osteoblasts per bone perimeter (N.Ob/B.Pm) measured by histomorphometry in male mice. *N* = 7–10 males per treatment. Significance was determined by multiple *t* tests with Holm-Šídák correction (**B**–**D**) and unpaired *t* test or Mann-Whitney test, as appropriate (**F**–**O**).

To test the functional contribution of p16^+^ myeloid-lineage cells toward age-related bone loss, we crossed our recently established *p16-LOX-ATTAC* mouse model (42) to mice carrying the *LysM-Cre* allele, which recombines specifically within myeloid cells (43). This model permits specific, inducible clearance of p16^+^ myeloid cells through administration of the AP20187 (AP) dimerizer, which activates the FKBP-Casp8 “suicide” transgene driven by the p16 promoter (similar to the previously developed *INK-ATTAC* model; refs. 6, 7) but only within cells expressing the Cre recombinase. To validate the efficacy and specificity of this model, we first treated 24-month-old *LysM-Cre* *p16-LOX-ATTAC* (*LysM-LOX*-*ATTAC*) mice with AP for 24 hours, after which bone marrow immune cells were phenotyped for senescence characteristics by CyTOF (Figure 2A). We found that monocytes/macrophages (CD14^+^ cells) were substantially reduced in both p16 expression and %p16^+^ cells compared with vehicle-treated mice, whereas B cells (CD19^+^) and T cells (CD3e^+^) were unaffected (Figure 2, B and C). Within CD14^+^ cells, we also observed a reduction in a number of senescence-associated proteins after targeted clearance of p16^+^ myeloid cells (Figure 2D).

We then tested the influence of p16^+^ myeloid cells toward age-related bone loss through long-term (4-month) treatment of old *LysM-LOX*-*ATTAC* mice with either vehicle or AP, followed by skeletal phenotyping by micro-computed tomography (μCT) (Figure 2E). In previous studies, we have demonstrated that this treatment regimen delays age-related bone loss in both global and osteocyte-specific p16^+^ cell clearance mouse models (41, 42). In female mice, we found no significant alterations in cortical thickness (Ct.Th) at either the femur diaphysis or metaphysis with clearance of p16^+^ myeloid cells (Figure 2, F and G). Additionally, we observed no changes in trabecular bone volume per total volume (BV/TV) at either the distal femur metaphysis or the lumbar spine (Figure 2, H and I). In male mice, although no changes were seen at the diaphysis or lumbar spine, metaphyseal Ct.Th and BV/TV were improved with AP treatment (Figure 2, J–M). However, we did not find any changes in osteoblast or osteoclast numbers on the endosteal bone perimeter (Figure 2, N and O). We also did not observe any improvements in mineral apposition or bone formation rates or any static parameters at either the periosteal or endosteal surfaces (Supplemental Figure 3, A–D). This was puzzling as there was still an effect of AP on cortical area at the metaphysis (Supplemental Figure 3E). However, we did find that AP-treated mice exhibited reduced metaphyseal cortical porosity (Supplemental Figure 3F), suggesting an intracortical effect that is independent of changes in periosteal or endosteal surfaces. It is also possible that changes in periosteal bone formation occurred early during the 4-month course of treatment but were not evident at the 4-month time point when our measurements were made. We did similar analyses at the trabecular compartment in the femur metaphysis (Supplemental Figure 3G) yet found no differences.

Males also demonstrated additional phenotypes related to body composition, with AP-treated mice exhibiting a preservation of total weight and lean mass over the course of treatment (Supplemental Figure 4, A–D). However, this did not appear to be due to a reduction in systemic inflammation, as serum levels of age-associated senescence- and inflammation-related factors were unchanged with AP treatment (Supplemental Figure 4E). To investigate tissue-specific effects in males, we performed bulk RNA-seq on skeletal muscle (quadriceps), spleen, and visceral fat (perigonadal) from male *LysM-LOX-ATTAC* mice to assess the extraskeletal effects on senescence burden. However, we did not observe any significant changes in SenMayo gene set enrichment or alterations in *Cdkn2a* expression with AP treatment in these nonskeletal tissues (Supplemental Figure 4, F and G). We also did not observe any significant differences in genes related to inflammation or tissue-specific physiology in either fat or muscle tissue (Supplemental Figure 4H).

These mixed skeletal effects led us to investigate any possible sex-specific cellular phenotypes within bone marrow myeloid cells (Supplemental Figure 5A). In our 24-hour-treated *LysM-LOX-ATTAC* mice, we observed that male mice demonstrated better clearance efficiency with AP treatment, suggested by a stronger magnitude of reduction in p16 expression and %p16^+^ cells than female mice (Supplemental Figure 5, I and J). However, no formal statistical interaction between mouse sex and treatment was observed through assessment by 2-way ANOVA (Supplemental Figure 5, I and J). When examining SASP profiles, it appeared that CD14^+^ cells from male mice exhibited a reduction in more factors than in female mice after p16^+^ cell clearance (Supplemental Figure 5, K and L), which likely resulted from the fact that CD14^+^ cells from male mice expressed higher levels of SASP proteins than females at baseline (Supplemental Figure 5, M and N). These results are perhaps not surprising, as age-related immune inflammation — specifically within myeloid cells — has been observed to be higher in males than females in both mice (44–46) and humans (47, 48). We continued these analyses in the Tabula Muris scRNA-seq dataset and analyzed bone marrow cells from 18-month-old mice (the oldest age containing males and females). We found that SenMayo expression was indeed higher in male *Cd14*^+^ cells (Supplemental Figure 6A), consistent with our CyTOF data. As a recent study found that estrogen signaling had antiinflammatory effects on macrophages in vitro, while androgens had no effect (49), we next investigated estrogen signaling pathways. In both total cells and *Cd14*^+^ cells, we found only minor expression of *Esr1* (encoding estrogen receptor α [ERα]) that was not higher in females and no detectable expression of *Esr2* (ERβ) or *Ar* (androgen receptor) (Supplemental Figure 6B). We also did not find an upregulation in any of 3 independent gene sets for estrogen signaling in *Cd14*^+^ cells (Supplemental Figure 6C), *Cd14*^+^SenMayo^+^ cells (Supplemental Figure 6D), or any myeloid cell clusters (Supplemental Figure 6E). This is consistent with our previous work (50), which showed 1) no effect of sex steroid deficiency (e.g., ovariectomy [OVX]/orchidectomy) on senescence markers in mouse bone, 2) no effect of estrogen treatment on senescence markers in elderly women, and 3) no effect of p16^+^ cell clearance on bone loss caused by OVX in mice. Thus, although our observation of increased SASP expression in aged myeloid cells from male mice aligns with recent observations by others in mice (44–46) and humans (47, 48), the influence of sex hormones on myeloid aging and senescence should be investigated in more detailed future mechanistic studies.

### p16^+^ myeloid cells are not stably suppressed after clearance in contrast with p16^+^ mesenchymal cells.

**Figure 3:**
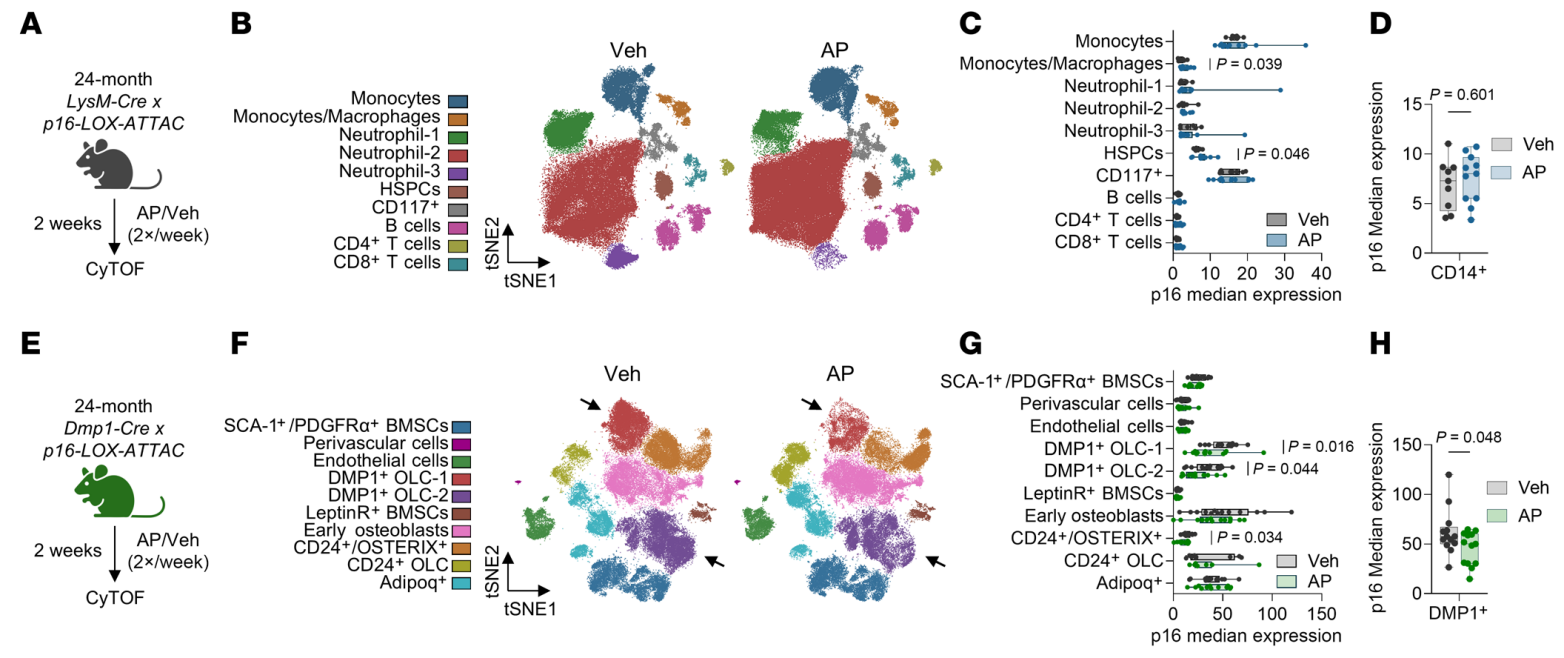
Targeted clearance of p16^+^ myeloid cells does not result in their long-term reduction as seen when targeting p16^+^ mesenchymal cells. (**A**) Schematic of 2-week treatment in 24-month-old LysM-LOX-ATTAC mice, which were treated twice weekly before bone marrow was isolated and analyzed by CyTOF. *N* = 9 vehicle (4 males, 5 females). *N* = 11 AP (5 males, 6 females). (**B**) t-SNE visualization of clustered bone marrow cells from treated LysM-LOX-ATTAC mice. (**C**) p16 median expression across all bone marrow cell clusters and (**D**) CD14^+^ myeloid cells. Significance was determined by unpaired *t* test. (**E**) Schematic of 2-week treatment in 24-month-old DMP1-LOX-ATTAC mice, which were treated twice weekly before mesenchymal cells were isolated and enriched from bone and marrow and analyzed by CyTOF. *N* = 13 vehicle (8 males, 5 females). *N* = 13 AP (7 males, 6 females). (**F**) t-SNE visualization of clustered mesenchymal cells from treated DMP1-LOX-ATTAC mice. Arrows point to DMP1^+^ OLC clusters, indicating clearance. (**G**) p16 median expression across all bone marrow cell clusters and (**H**) DMP1^+^ osteocytes. Significance was determined by unpaired *t* test.

Given these limited effects of p16^+^ myeloid cell clearance on age-related bone loss, we next sought to understand the potential underlying mechanisms for why these effects were so different from either global (41) or mesenchymal (osteocyte-specific, ref. 42) senescent cell clearance. In models of global senescent cell clearance, we have previously demonstrated that a 2-week treatment of *INK-ATTAC* mice where the mice are harvested 96 hours following the last dose of vehicle or AP led to observable reductions in both senescent cell numbers and p16/SASP expression in mesenchymal populations by CyTOF (17). Thus, we used a similar experimental design and treated 24-month-old *LysM-LOX-ATTAC* mice with vehicle/AP for 2 weeks and analyzed bone marrow immune populations by CyTOF 96 hours following the last dose of vehicle/AP to assess effects on senescent cell burden (Figure 3A). Surprisingly, in *LysM-LOX-ATTAC* mice, we found no differences in immune cell population abundances (Figure 3B and Supplemental Figure 7, A and B) or reductions in p16 median expression (Figure 3C) from mice treated with AP for 2 weeks and harvested 96 hours after the last AP dose. When segregated by sex, there were still no significant changes in p16 expression in immune cell populations (Supplemental Figure 7C). Additionally, we observed no reduction in p16 expression in CD14^+^ cells in all mice (Figure 3D) or in a sex-specific manner (Supplemental Figure 7D) and observed no changes in any other manually gated myeloid population (Supplemental Figure 7E). To ensure that the *LysM-LOX*-*ATTAC* model was not producing aberrant results and to further test our findings in myeloid cells, we performed the same treatment and CyTOF analysis on 24-month-old *INK-ATTAC* mice (global senescent cell clearance model) treated with vehicle/AP for 2 weeks and harvested 96 hours following the last vehicle/AP dose (Supplemental Figure 8, A–C). We found that, in contrast with our previously observed changes in mesenchymal cells (17), we still did not observe any alterations in cell abundance or p16 expression in immune cell populations after 2 weeks of AP treatment, even when segregating by sex (Supplemental Figure 8, D–G).

To compare these findings with mesenchymal cells, we crossed our *p16-LOX-ATTAC* mice with mice containing the *Dmp1-Cre* allele, which permits specific targeting of p16^+^ osteocytes (42, 51). These mice were aged to 24 months, then treated with either vehicle or AP for 2 weeks and harvested 96 hours following the final vehicle or AP dose — identical to our regimen in *LysM-LOX-ATTAC* mice — and analyzed by CyTOF (Figure 3E and Supplemental Figure 9, A and B). In contrast with myeloid cells, we found significant reductions in p16 expression within both clustered DMP1^+^ osteolineage cells and total DMP1^+^ cells after 2 weeks of AP treatment (Figure 3, F–H). We observed substantial reductions in %p16^+^ cells within DMP1^+^ osteolineage clusters as follows (mean ± SD): DMP1^+^ OCL-1: Veh = 5.06% ± 6.26%, AP = 0.645% ± 0.838% (~87% reduction). DMP1^+^ OCL-2: Veh = 0.518% ± 0.510%, AP = 0.141% ± 0.243% (~73% reduction) (Supplemental Figure 9C).

Collectively, our analysis of the skeletal consequences of mesenchymal versus myeloid p16^+^ senescent cell clearance demonstrated that the skeletal effects of senescent myeloid cell clearance are modest as compared with our previous findings following either global (41) or mesenchymal (osteocytic; ref. 42) senescent cell clearance. In direct comparisons, it appears that mesenchymal senescent cells remain reduced for up to 96 hours (and perhaps longer) following their clearance, whereas myeloid p16^+^ cells are reduced by 24 hours after clearance but appear to rapidly repopulate by 96 hours. This is perhaps derived from the presumptive lifespan and generation rate of each cell type, as myeloid-lineage cell half-lives are estimated to be as low as 2–3 days (33, 52), while osteocytes and other terminally differentiated mesenchymal cells have half-lives of months to years (53). Thus, it may be that mesenchymal senescence is evident as an accumulation of long-lived cells, where their clearance leads to stably reduced senescence burden, while immune senescence is short-lived, with these cells repopulating at a much faster rate than mesenchymal cells.

### Myeloid cells display lower absolute levels of senescence markers than mesenchymal cells.

**Figure 4:**
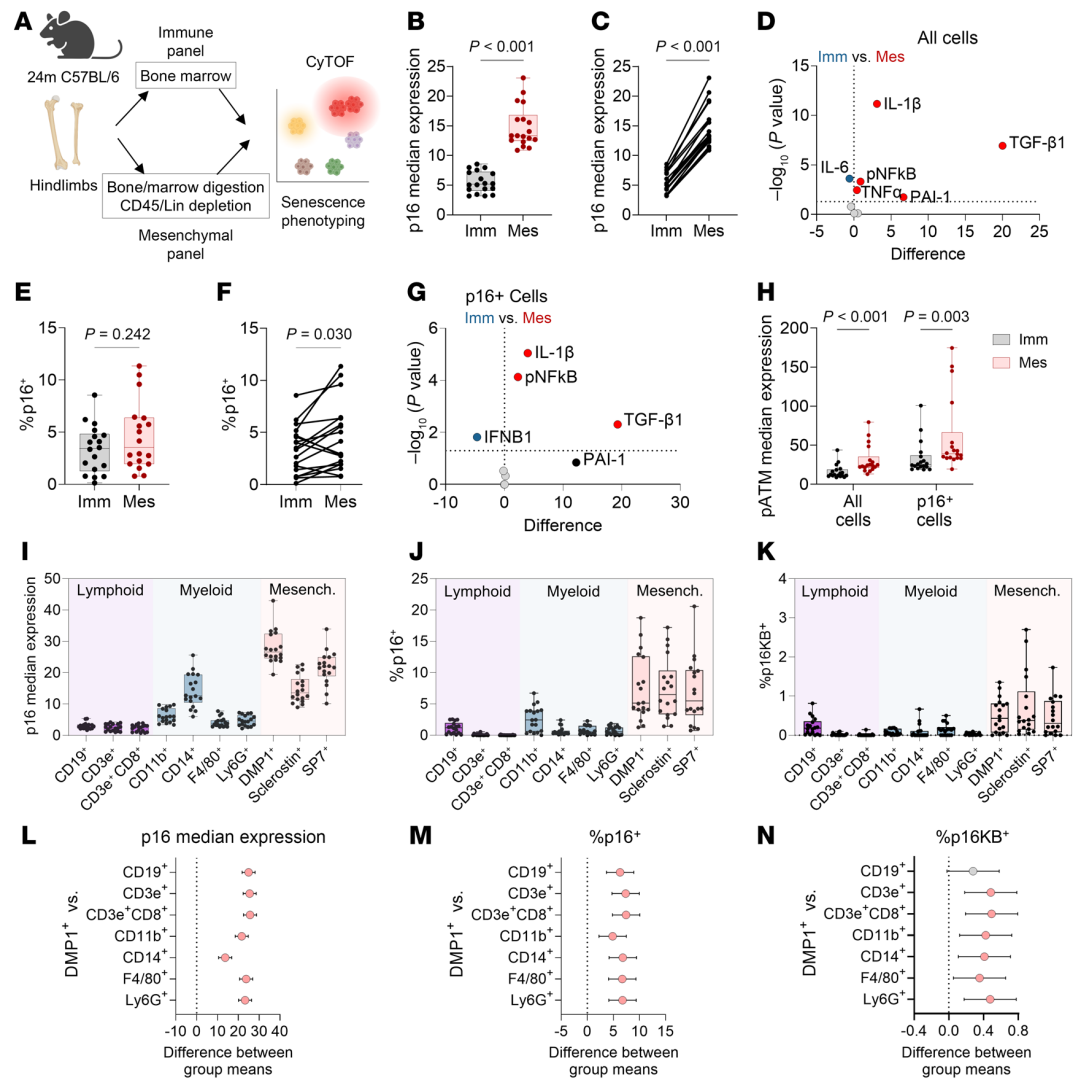
Mesenchymal cells exhibit higher absolute levels of senescence signatures than immune cells. (**A**) Schematic outlining workflow of paired CyTOF analysis of both immune and mesenchymal cells from the same bone sample in 24-month-old C57BL/6N wild-type (WT) mice. *N* = 18 mice total (9 males, 9 females). (**B**) p16 median expression of all immune (Imm) or mesenchymal (Mes) cells, shown as paired samples (**C**). (**D**) Volcano plot of SASP marker differences between immune and mesenchymal cells. (**E**) %p16^+^ cells in immune or mesenchymal cells, shown as paired samples in **F**. (**G**) Volcano plot of SASP marker differences between p16^+^ cells in either immune or mesenchymal compartments. (**H**) pATM expression in all cells and p16^+^ cells between immune or mesenchymal compartments. (**I**–**K**) p16 median expression, %p16^+^ cells, and %p16^+^Ki67-BCL2^+^ (p16KB) cells in various lymphoid, myeloid, and mesenchymal cell populations. Mesench., mesenchymal. (**L**–**N**) One-way ANOVA results with Bonferroni’s correction on corresponding data from **I**–**K** showing differences in group means of DMP1^+^ cells versus various immune cell types, with error bars as 95% confidence intervals. Significance was determined by unpaired *t* test or Mann-Whitney test, as appropriate (**B** and **E**); Wilcoxon’s matched pairs signed-rank test (**C** and **F**); or multiple *t* tests with Holm-Šídák correction (**D** and **G**).

To further contrast immune and mesenchymal cells expressing senescence features, we performed a paired-analysis study to assess senescence burden directly between these 2 compartments within the same mouse (Figure 4A). Briefly, we isolated hind limbs from 24-month-old WT (C57BL/6N) mice and performed CyTOF analysis on both bone marrow immune cells and CD45^–^Lin^–^ mesenchymal cells from digested bone and marrow. When examining total cells in each population, we found that the absolute level of p16 expression was 170.2% higher (±16.9 SD, *P* < 0.0001) in mesenchymal cells than immune cells (Figure 4B), and paired analyses demonstrated that this trend was true in every single mouse (Figure 4C). Furthermore, mesenchymal cells exhibited higher levels of SASP proteins than immune cells (Figure 4D). When assessing percentages of p16^+^ cells, we did not observe any differences in overall values (Figure 4E); however, we found with paired analyses that on a per-mouse basis, there was a higher percentage of p16^+^ cells in mesenchymal versus immune compartments (Figure 4F). Moreover, aged mesenchymal p16^+^ cells demonstrated higher levels of SASP proteins than aged immune p16^+^ cells (Figure 4G). We also observed differential expression of the DNA damage marker pATM, which was higher in both total and p16^+^ mesenchymal cells (Figure 4H).

We next sought to directly compare different immune and mesenchymal compartments through cell type analyses. Using manual gating, we specified common cell types within immune (B cells [CD19^+^], T cells [CD3e^+^], cytotoxic T cells [CD3e^+^CD8^+^], myeloid-lineage [CD11b^+^], monocytes/macrophages [CD14^+^], macrophages [F4/80^+^], neutrophils [Ly6G^+^]) and mesenchymal (mature osteoblasts/osteocytes [DMP1^+^], osteocytes [Sclerostin^+^], osteoblasts [SP7/Osterix^+^]) compartments (Figure 4, I–K). When contrasting cell types from all 3 compartments (i.e., lymphoid, myeloid, and mesenchymal), it was clear that mesenchymal cells exhibited the highest level of p16 expression, percentage of p16^+^ cells, and percentage of p16^+^Ki67^–^BCL2^+^ cells (Figure 4, I–K), which we previously found to be more specific for age-related senescence within bone mesenchymal cells (17). This was particularly true for DMP1^+^ cells (Figure 4, L–N), which notably displayed substantially higher levels of p16 expression than CD14^+^ cells (27.99 ± 5.37 vs. 14.26 ± 5.31) and CD11b^+^ cells (6.315 ± 2.22) and over a full order of magnitude higher than CD3e^+^CD8^+^ cytotoxic T cells (2.502 ± 1.114) (Figure 4I). Similarly, other osteolineage cells were also significantly higher in these parameters compared with nearly all lymphoid and myeloid populations (Supplemental Figure 9, D–F).

### Myeloid cells exhibit aging-independent DNA damage and altered response to senescence induction.

**Figure 5:**
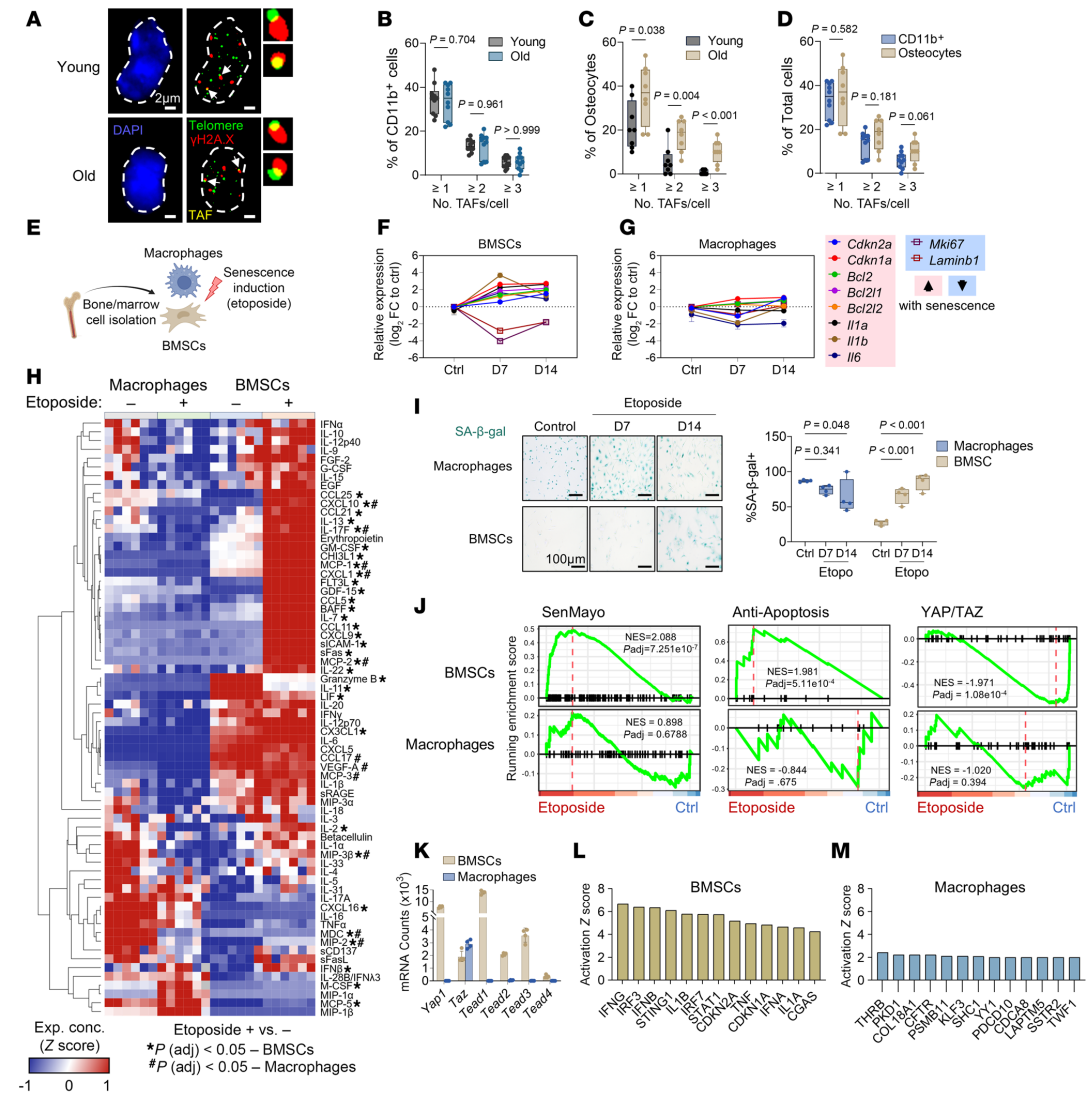
Myeloid cells exhibit divergent senescence characteristics and altered response to senescence induction compared with mesenchymal cells. (**A**) Representative images of telomere-associated foci (TAF; white arrows) on CD11b^+^ bone marrow cells from young (6-month-old) and old (24-month-old) wild-type (WT) C57BL/6N mice. *N* = 10 per age. (**B**) Quantification of %TAF^+^ CD11b^+^ cells subset by the number of TAF observed per cell. (**C**) Quantification of %TAF^+^ osteocytes from a previous study from our laboratory (Farr et al., ref. 42; *N* = 8 per age). (**D**) Comparisons between %TAF^+^ subsets between CD11b^+^ cells and osteocytes. Significance was determined by unpaired *t* test or Mann-Whitney test, as appropriate. (**E**) Schematic of in vitro study comparing molecular responses to senescence induction by etoposide between bone marrow–derived macrophages (BMDMs; macrophages) and bone marrow stromal cells (BMSCs). (**F** and **G**) qPCR results from senescence induction experiments, demonstrating relative mRNA levels of senescence-associated genes (see Supplemental Figure 10 for individual gene plots and statistics). *N* = 4–9 per cell type. (**H**) Cytokine analysis of conditioned media from control and D14 etoposide-treated BMSCs and macrophages. Significance was determined by multiple unpaired *t* tests with Holm-Šídák correction of etoposide treated versus control for each cell type. (**I**) SA-β-gal staining of BMSC and macrophage cultures and quantification. *N* = 4 per cell type. Significance was determined by 2-way ANOVA with Tukey’s multiple comparisons test. (**J**) Gene set enrichment analysis (GSEA) plots from RNA-seq data of control and D14 etoposide-treated BMSC and macrophage samples for the following gene sets: SenMayo, antiapoptosis (see Supplemental Table 3), and YAP/TAZ activity (CORDENONSI_YAP_CONSERVED_SIGNATURE). *N* = 3–4 per treatment, per cell type. (**K**) Raw mRNA counts of YAP/TAZ-TEAD related genes. (**L** and **M**) Predicted upstream activators in BMSCs (cGAS/STING and senescence-related) and macrophages (all) (complete data in Supplemental Table 4). SA-β-gal, senescence-associated β-galactosidase; cGAS/STING, cyclic GMP–AMP synthase (cGAS)–stimulator of interferon genes (STING).

We next sought to characterize myeloid and mesenchymal cells within the context of senescence development through histological and ex vivo approaches. As senescence is known to be induced by DNA damage (54), we assessed myeloid-lineage (CD11b^+^) cells isolated from young and old murine bone marrow for DNA damage using the assay for telomere-associated foci (TAF) (55). We found that while CD11b^+^ cells do indeed display TAF (Figure 5A), neither the frequency of TAF^+^ cells, nor the number of TAF per cell, is changed with age (Figure 5B). This is in direct contrast with osteocytes, which were previously shown to display substantially increased TAF with age, specifically ≥3 TAF per cell (42) (Figure 5C). When comparing the 2 populations in old mice, there was a nonsignificant (*P* = 0.0614) trend toward a higher frequency of cells with ≥3 TAF in osteocytes than in CD11b^+^ cells (Figure 5D). To investigate this discrepancy in DNA damage accrual, we performed bulk RNA-seq on bone marrow monocytes (isolated by magnetic activated cell sorting [MACS] with the mouse Monocyte Isolation Kit [Miltenyi Biotec]) from young and old mice and analyzed these cells alongside previously collected samples of similarly aged mesenchymal enriched bone samples (30) (National Center for Biotechnology Information [NCBI] Gene Expression Omnibus [GEO] GSE199493) (Supplemental Figure 10A). With age, monocytes were significantly enriched in genes associated with DNA repair, while mesenchymal cells were not (Supplemental Figure 10, B and C).

To further understand how DNA damage influences senescence in myeloid cells versus mesenchymal cells, we performed senescence induction using etoposide treatment on primary mouse bone marrow–derived macrophages (BMDMs) and mesenchymal bone marrow stromal cells (BMSCs) in side-by-side in vitro experiments (Figure 5E). Of note, we were unable to induce replicative senescence in BMDMs, as these cells typically died after approximately 2 weeks in culture, and while BMSCs survived H_2_O_2_ treatment and went on to develop a senescent phenotype, BMDMs did not survive the H_2_O_2_ treatment. Thus, our experiments focused on using etoposide to induce DNA damage in these cells. In contrast with BMSCs, macrophages did not develop a transcriptional profile of senescence development following etoposide treatment (Figure 5, F and G, provide composite summaries; Supplemental Figure 10, D–N, provide individual gene plots and statistics). Specifically, although macrophages did upregulate *p16^Ink4a^* and *p21^Cip1^* expression by day 14 after etoposide exposure (albeit to a much lesser extent than BMSCs), they did not significantly upregulate mRNAs encoding genes regulating apoptosis resistance (*Bcl2*, *Bcl2l1* [BCL-XL], and *Bcl2l2* [BCL-W]) or SASP (*Il1a*, *Il1b*, *Il6*). Etoposide-treated macrophages also did not display reductions in *Laminb1* or *Mki67*, which are additional hallmarks of senescence (56). We next performed cytokine analysis on conditioned media from control and etoposide-treated BMSCs and macrophages to identify secreted regulators of bone remodeling (Figure 5H and Supplemental Figure 9, O–T). BMSCs had a large number of proteins upregulated, including senescence markers GDF-15, CXCL9, and MCP-1, while the only differentially expressed proteins in macrophages were downregulated after etoposide treatment, suggesting that macrophages become less secretory following DNA damage. Also, several factors known to promote bone resorption (IL-1α, TNF-α, and macrophage inflammatory protein 3β) are expressed in untreated macrophages at comparable or higher levels than senescent BMSCs, suggesting that any factors released by myeloid cells that affect bone remodeling are probably released independent of senescence. Furthermore, macrophages stained nonspecifically for senescence-associated β-galactosidase (SA-β-gal), as observed by others (25, 31), while BMSCs exhibited an expected increase in percentage of SA-β-gal^+^ cells after etoposide treatment (Figure 5I). Using bulk RNA-seq and gene set enrichment analyses, we found that etoposide-treated BMSCs exhibited a robust enrichment in the SenMayo senescence gene set, while macrophages did not (Figure 5J). Additionally, enrichment of antiapoptotic genes — another hallmark of senescence — was found only in BMSCs treated with etoposide, not in macrophages (Figure 5J). Similar patterns were observed in vivo, as freshly isolated bone marrow monocytes demonstrated limited upregulation of *Cdkn2a* mRNA counts with age, in contrast with the robust upregulation in mesenchymal enriched samples, while neither cell type increased in *Cdkn1a* expression with age, consistent with our previous work in mesenchymal cells by CyTOF (17) (Supplemental Figure 8, O and P).

Etoposide-treated BMSCs also showed a loss of YAP/TAZ activity, a mechanosignaling pathway known to prevent aging and senescence in mesenchymal cells (Figure 5J) (26). Loss of YAP/TAZ upregulates cyclic GMP–AMP synthase (cGAS)–stimulator of interferon genes (STING) signaling, a cytosolic DNA sensing pathway that triggers an inflammatory response, which drives inflammation and the SASP (26). Interestingly, YAP/TAZ signaling was not significantly altered in macrophages (Figure 5J). Upon further investigation, this appears to be due to a near absence of baseline expression of mRNA transcripts encoding the YAP/TAZ-TEAD complex in macrophages (Figure 5K), other than TAZ, which has been shown to be largely redundant without YAP expression (57). Accordingly, recent studies have linked macrophage inflammation to activation/upregulation of YAP/TAZ (58, 59), highlighting the pleiotropic roles of senescence-related proteins in immune versus mesenchymal cell physiology. To identify driving forces behind this cell-dependent response, we used the Upstream Regulator Analysis tool within the Ingenuity Pathway Analysis program (Figure 5, K–M, and Supplemental Table 4). This revealed a substantial number of predicted upstream regulators in BMSCs treated with etoposide, predominantly consisting of activating factors belonging to the cGAS/STING signaling pathway (Figure 5L) (e.g., cGAS, STING1, IFNG/B, IRF3). This was not true in macrophages, which displayed a smaller number of unrelated gene programs with lower activation scores (Figure 5M and Supplemental Table 4). This suggests that the inability of macrophages (and other immune cells, based on the complementary findings of Sladitschek-Martens et al., ref. 26) to orchestrate the senescence program like mesenchymal cells may be due to an absence of mesenchymal cell–specific signaling patterns that drive senescence, such as YAP/TAZ. Collectively, these results demonstrate a discordance in the expression of senescence markers and functional senescence in myeloid-lineage cells, providing evidence that myeloid-lineage cells do not respond to senescence induction similar to mesenchymal cells.

## Discussion

In this study, we investigated the senescence characteristics of bone marrow immune cells during murine aging. We found that among the immune cell populations in the bone marrow of aged mice, myeloid cells exhibited the most robust senescence signals, but their senotype differed substantially from that of senescent mesenchymal cells, which have been studied in far greater detail in bone (17, 41, 42) and in other tissues (11, 13, 18). Specifically, while myeloid cells expressed the highest levels of p16, p21, and SASP factors among bone marrow immune cell populations, these levels were still considerably lower than those observed in aged mesenchymal cells. Moreover, p16^+^ myeloid cells repopulated rapidly following genetic clearance in vivo, likely explaining the limited skeletal benefits of p16^+^ myeloid cell clearance we observed, which contrasts with the substantial beneficial skeletal effects of mesenchymal senescent cell clearance we previously demonstrated (41, 42). These differences in the kinetics of mesenchymal versus myeloid senescent cell generation and clearance are summarized in Supplemental Figure 10W. In addition to our in vivo findings, we also found that myeloid cells failed to express the classical senescence phenotype exhibited by mesenchymal cells following senescence induction in vitro. Thus, the senotype of aged bone marrow myeloid cells appears to be that of partial, rather than complete, deep senescence. This difference between aged immune and mesenchymal cells was alluded to by the work of Sladitschek-Martens et al. (26), but our analysis indicates that aged myeloid and mesenchymal cells differ substantially in their ability to develop the classical senescence phenotype as described for mesenchymal cells (1, 3). As myeloid cells greatly outnumber mesenchymal cells in the bone microenvironment, this suggests that the sheer number of p16^+^ myeloid cells is likely much higher than the number of p16^+^ mesenchymal cells; however, their clearance has a less substantial effect on age-related bone loss. This further underscores the limited senescence phenotype of p16^+^ myeloid cells and provides evidence toward a rejection of the hypothesis that all p16^+^ cells have an equally damaging effect on tissue physiology. A possible new hypothesis may be that senescence in cells that are primarily responsible for tissue maintenance (e.g., osteolineage cells in bone) has the greatest effect on age-related decline of said tissue, rather than paracrine SASP effects of nearby cells (e.g., immune cells). To our knowledge, this hypothesis is untested and would warrant investigation in other tissues in future studies.

The mechanisms that underlie the differences in senotypes between myeloid- and mesenchymal-lineage cells remain unclear but several possibilities exist. First, these differences may be due to the inherent lifespan and generation rate of cells within each lineage. A majority of immune cells are short-lived, while mesenchymal cells are generally long-lived. It may be that both lineages respond to cellular stress with the initiation of a senescence program, but immune cells may not reach the state of deep senescence observed in mesenchymal cells due to either programmed cell death or clearance. Second, our finding that while both myeloid and mesenchymal cells demonstrate evidence of DNA damage, the upregulation of DNA repair pathways with age in myeloid cells we observed may allow these cells to avoid senescence, and this possibility certainly warrants further study. Finally, certain pathways that are highly active in mesenchymal cells, such as YAP/TAZ (60), may regulate senescence development in a manner independent of immune aging, as shown by Sladitschek-Martens et al. (26). Importantly, our findings are entirely consistent with work from this group (26), which demonstrated that YAP/TAZ activity declined with aging in mesenchymal, but not immune cells, and that the decline in YAP/TAZ was critically linked to expression of the full senescent phenotype in mesenchymal cells, including loss of lamin B1 and the production of a SASP. Indeed, maintenance of YAP/TAZ signaling in mesenchymal cells markedly attenuated the senescent phenotype of these cells (26).

In our in vitro studies, we present data using etoposide to induce senescence in BMSCs and macrophages. As noted, we also attempted alternative approaches, including exposure to H_2_O_2._ However, the BMDMs did not survive the H_2_O_2_ treatment and died shortly thereafter, whereas the BMSCs survived and went on to develop a senescent phenotype. The reason(s) why BMSCs survive DNA damage due to etoposide as well as H_2_O_2_ but the myeloid cells are only able to survive etoposide but not H_2_O_2_ are unclear but do point further to important differences between these cells in terms of their ability to develop a senescence phenotype.

Our findings with regard to the senotype of aged myeloid cells is also consistent with recent work by Ashraf et al. indicating that senescence may not be a simple on/off state (61). Using detailed in vitro analyses of cells following the induction of DNA damage, these investigators demonstrated a spectrum of cell fates ranging from quiescence to a gradient of senescence phenotypes. Consistent with this, we (17) and others (13) have demonstrated the presence in vivo of mesenchymal cells expressing features of senescence, including upregulation of p16 and p21 as well as evidence of DNA damage and a SASP, that are not growth arrested. In this context, aged myeloid cells appear to express a partial senescence phenotype partway through the spectrum of senescence described by Ashraf et al. (61).

Although distinct from mesenchymal cells, the senotype displayed by myeloid cells did indeed show relevant levels of inflammation, while contributing to age-related bone loss, albeit to a low level and only in males. Others have shown that aged bone marrow macrophages drive paracrine senescence that contributes to aging of various tissues (62), and it has been shown both in mice and in humans that males demonstrate higher levels of immune inflammation with age (44–46, 48, 63), particularly in monocytes (47). Our work suggests that this may be at least partially driven by myeloid cells expressing markers of senescence; however, similar to others, we could not identify effects of sex hormones or receptors on this phenomenon. If we were to speculate, it may be that female sex hormone signaling is protective against immune aging, but the signaling may be through other receptors. One example would be the action of estrogen on GPER1, which acts to suppress IFN-γ signaling and inflammation (64, 65). In addition, the higher levels of immune inflammation with age in male mice may explain the greater susceptibility of p16^+^ myeloid cells from male mice to senolytic clearance with the AP drug and lack of a significant effect on these cells in female mice, although further studies are needed to evaluate these sex differences.

One clinically relevant feature of these distinct senotypes is the observational point where reductions in senescence burden can be captured. As illustrated in Supplemental Figure 10W, our work reveals that clearance of p16^+^ myeloid cells is evident 24 hours after senolytic clearance but not detectable after 96 hours of senolytic treatment. This contrasts with clearance of mesenchymal cells, which can reproducibly be observed after 96 hours (and perhaps longer) of treatment both in this work and previously (17). Understanding these kinetics of senescent cell clearance may be particularly valuable in the translation of senolytics to clinical trials, as the ability to demonstrate reductions in senescent cell burden in tissue samples will likely be necessary to link to any tissue-level phenotypic effects. Moreover, if it can be determined which senescent cell type is perpetuating the disease (e.g., mesenchymal or immune), altered treatment regimens can be established to both maximize efficacy (more frequent dosing for short-lived senescent immune cells) and limit redundancy (less frequent dosing for long-lived senescent mesenchymal cells). Thus, it will be of considerable importance to further investigate the senotypes of cells within aging tissues to establish reproducible methods for both targeting and subsequent observation of clearance of disease-driving senescent cells.

There are several limitations to this study, one of which is that the focus of this work was immune cells only within the bone marrow. As bone marrow aging has widespread organismal effects (66–68), it remains to be tested whether these myeloid-lineage senotypes influence aging of hematopoietic stem cells or exist in the circulation and promote the decline of tissues outside the skeleton. Furthermore, the myeloid cells phenotyped in this setting may differ from tissue-resident myeloid cells, such as microglia (69–71) or alveolar macrophages (9, 10), which may display their own unique senotypes due to the long-lived nature of tissue-resident macrophages (72). We also recognize that additional strategies, such as more frequent dosing with AP in the *LysM-LOX-ATTAC* mice, could be used to test whether that results in more substantial skeletal changes. However, our goal was to provide a detailed characterization of the differing senotypes of bone marrow myeloid versus mesenchymal cells rather than fully characterize the best therapeutic approach to target senescent-like myeloid cells. Finally, our work focused on myeloid cells because at least in the bone marrow, these cells exhibited the highest expression of p16 and SASP factors, but further studies are clearly needed to define the senotypes of bone marrow and other tissue-resident T and B cells. Nevertheless, we expect this work to serve as a basis for future investigation of these issues, and mapping senotypes of various aging tissues, directly comparing mesenchymal to immune cells within the same tissue as done here, should advance our understanding of the complexities of senescent cell types across tissues.

In summary, using single-cell methods and cell-specific senolytic clearance models, we find that bone marrow myeloid-lineage cells display features of senescence substantially different from bone mesenchymal cells and have a limited contribution to skeletal aging. Collectively, our work provides evidence supporting the hypothesis that bone marrow myeloid cells do not develop the characteristic “Hayflick” senescence originally described in mesenchymal cells (1, 3, 26). Our findings also point to the need to not simply extend the concept of cellular senescence from mesenchymal to immune cells but rather carefully evaluate the senotypes of aged immune versus mesenchymal cells across tissues.

## Methods

### Sex as a biological variable.

In accordance with NIH guidelines (73), we studied both female and male mice in this study. Where appropriate, males and females were analyzed separately due to known or newly discovered sex-specific effects. Moreover, as recently recommended (74), we performed 2-way ANOVA tests on several important parameters (Supplemental Figure 5) to test for possible interactions between sex and treatment on our endpoints.

### Animals.

Mice were housed in ventilated cages and maintained within a pathogen-free, accredited facility with constant temperature (25°C), 30%–70% humidity, a 12-hour light/dark cycle, and access to food and water ad libitum. Mice used in this study included *C57BL/6N* WT, *p16-LOX-ATTAC* (42), *INK-ATTAC* (7), *LysM-Cre* (43), and *Dmp1-Cre* (75) mice. In *p16-LOX-ATTAC* and *INK-ATTAC* experiments, mice were randomized by both spine bone mineral density and weight for injection with either vehicle (4% ethanol, 10% polyethylene glycol 400, and 2% Tween) or 10 mg/kg AP20187 (MedChemExpress catalog HY-13992) dissolved in vehicle, administered by intraperitoneal injection twice weekly. For each experiment, mouse numbers, ages, and sexes are indicated in the figure legend.

### CyTOF cell isolations.

Mice were euthanized according to approved and standardized IACUC protocols. For the isolation of hematopoietic/immune cells, femurs and tibias were isolated, cleaned of soft tissue, and cut at both ends at the metaphyses. Marrow was centrifuged out of both diaphyses and metaphyses at 10,000 xg for 10 seconds into a collection tube and treated with RBC lysis buffer (Thermo Fisher Scientific). For mesenchymal cells, diaphyses and metaphyses were gently crushed and digested in 300 U/mL of Collagenase IA (Sigma) diluted in MEM-α (Thermo Fisher Scientific) 3 times for 25 minutes each. Cells were then treated with RBC lysis buffer and depleted of cells expressing hematopoietic lineage markers (CD5, CD45R [B220], CD11b, Gr-1 [Ly-6G/C], 7–4, and Ter-119) using the Lineage Cell Depletion Kit (Miltenyi Biotec) and MACS.

### CyTOF antibodies.

Metal-conjugated antibodies used for all CyTOF analyses are summarized in Supplemental Table 1. Antibodies were either preconjugated (Standard BioTools) or conjugated in-house to isotopically enriched lanthanide metals using the MaxPAR antibody conjugation kit (Standard BioTools). Labeled antibodies were stored in PBS supplemented with glycerol, 0.05% BSA (Sigma), and 0.05% sodium azide at 4°C. All antibodies were tested with control beads as well as positive and negative control cells. A detailed validation of key CyTOF antibodies used in our panels (e.g., p21, p16, others) was performed previously (17).

### CyTOF staining and sample processing.

Conjugated antibody concentrations were measured by absorbance at OD_280_ nm and normalized to a 5 μg/μL stock concentration. Isolated cells were resuspended in 1 mL of Cell Staining Buffer (CSB) (Standard BioTools) and incubated with 0.5 μm Cisplatin solution (Standard BioTools) for 5 minutes in PBS. Samples were then washed twice with CSB and stained with an antibody master mix of the entire phenotyping panel (Supplemental Table 1) in CSB. Samples were stained at room temperature for 45 minutes, washed twice, and fixed with 2% paraformaldehyde (Standard BioTools) in PBS. Samples were then resuspended in 30 nM intercalation solution (Standard BioTools) and incubated overnight at 4°C. The following morning, cells were washed with PBS and resuspended in a 1:10 solution of calibration beads and cell acquisition solution (Standard BioTools) at a concentration of 0.5 × 10^6^ cells/mL. Samples were filtered through a 35 μm blue cap tube (Falcon) prior to data acquisition. Samples were loaded onto a Helios CyTOF system (Standard BioTools) and acquired at a rate of 200–400 events/s. Using the CyTOF software (Version 6.7.1014), data were collected as FCS files, and intrafile signal drift was normalized to acquired calibration bead signal.

### CyTOF data analysis.

Cleanup of cell debris — including removal of debris, beads, dead cells, and doublets (Supplemental Figure 2A) — was performed using Cytobank software (76, 77). Dimensionality reduction was achieved using viSNE (5,000 iterations, 100 perplexity, 0.5 theta), which is based on the t-SNE algorithm (78). FlowSOM clustering was performed within Cytobank (hierarchical consensus, 10 iterations), and cluster labels were assigned by expression of identifying markers curated from established literature, with relative marker intensities per cluster visualized by heatmap. FCS files were exported, concatenated in R, then re-uploaded for visualization of merged populations. Quantified values were exported to GraphPad Prism 10 to construct plots and perform statistical analyses.

### scRNA-seq analysis.

Single-cell transcriptomic analysis of murine bone marrow mononuclear cells across aging was performed on a previously published dataset from the *Tabula Muris Senis* (36). Files were downloaded in H5AD format from FigureShare and converted into a single-cell object in R using the Seurat v4 package (79). Samples from mice aged 24 and 30 months old were subset from the original data to specifically study cells from old animals. Visualization was performed using the SCpubr (80) and Nebulosa (81) packages.

### Skeletal and body composition assessments.

For treatment randomization, baseline areal bone mineral density (g/cm^2^) of the lumbar spine (L1–L4) was measured in vivo by dual-energy x-ray absorptiometry using a PIXImus densitometer (software v1.44.005; Lunar Corp.). Body mass (g) was recorded at baseline and at monthly points on all treated mice. At baseline and study endpoint, body composition (total body lean and fat mass) was assessed in mice by quantitative Echo magnetic resonance imaging (EchoMRI-100). All μCT imaging and analyses were performed on bones after euthanasia ex vivo. Quantitative measures of bone microarchitectural parameters of the right femur (distal metaphysis and midshaft diaphysis) and lumbar vertebrae (L5) were performed using the following scanner settings: 55 kVp, 10.5 μm voxel size, 21.5 diameter, 145 mA, 300 ms integration time. Trabecular BV/TV was measured at the distal metaphysis (100 slices) of the right femur and the lumbar spine (200 slices). In addition, Ct.Th (mm) was assessed at the distal metaphysis (50 slices) and mid-diaphysis (50 slices) of the right femur.

### Bone histomorphometry.

For dynamic histomorphometric analyses, mice were injected subcutaneously with Alizarin-3-methyliminodiacetic acid (0.1 mL/animal, 7.5 mg/mL) and calcein (0.1 mL/animal, 2.5 mg/mL) on days 9 and 2, respectively, before euthanasia. After μCT scanning, lumbar vertebrae and the right femur were embedded in methyl methacrylate. Bone sectioning and histomorphometry were performed as previously described by our laboratory (82).

### FACS and MACS.

To purify myeloid-lineage cells for histological staining, femurs and tibias were isolated, cleaned of soft tissue, and cut at both ends at the metaphyses. Marrow was centrifuged out of both diaphyses and metaphyses into a collection tube and treated with RBC lysis buffer. Cells were stained in FACS buffer (0.5% BSA in PBS) at 4°C in the dark first with FcR Blocking Reagent (Miltenyi Biotec) for 10 minutes, followed by Alexa Fluor 647 anti-CD11b antibody (BioLegend; catalog 101218) at 1:500 dilution for 15 minutes. Cells were then stained with SyTOX Blue (Thermo Fisher Scientific) at 1:5,000 dilution for 5 minutes at 4°C, spun down at 300*g* for 5 minutes at 4°C, resuspended in FACS buffer at a concentration of 1 × 10^7^ cells/mL, and analyzed on a FACSAria II (BD Biosciences). Single-color and unstained controls were used for compensation and to set gates. Monocytes isolated for RNA-seq were prepared from marrow as above but purified using negative selection with MACS with the mouse Monocyte Isolation Kit from Miltenyi Biotec.

### TAF staining.

TAF staining was performed on CD11b^+^ cells isolated by FACS, as described above. Details regarding the TAF staining have been previously described (83).

### Cell culture.

Primary mouse BMSCs were generated from 6-month-old C57BL/6N mice by digesting freshly dissected femurs and tibias 3 times for 25 minutes each in collagenase, by RBC lysis, and by MACS depletion of hematopoietic cells, as described above for CyTOF preparations. BMSCs were plated in 75 cm^2^ flasks in BMSC media (1 g/L glucose DMEM [Thermo Fisher Scientific] + 10% FBS [Gemini Bio-Products] + 1× Antibiotic/Antimycotic [Thermo Fisher Scientific] + 1× Gentamicin [Sigma]) and expanded in a hypoxic incubator (3% O_2_, 5% CO_2_, 37°C). BMDMs were generated by differentiating monocytes isolated from the bone marrow (femurs and tibiae) of 6-month-old C57BL/6N mice. Specifically, marrow cells were treated with RBC lysis buffer (as described above for CyTOF preparations), and monocytes were isolated via MACS using the mouse Monocyte Isolation Kit. Monocytes were plated in BMDM media (4.5 g/L glucose DMEM [Thermo Fisher Scientific] + 10% FBS [Gemini Bio-Products] + 1× Antibiotic/Antimycotic [Thermo Fisher Scientific] + 1× Gentamicin [Sigma]) plus 20 ng/mL M-CSF (PeproTech) for 7 days to obtain BMDMs. BMSCs and BMDMs were seeded at 10,000 cells/cm^2^ in 6-well plates and treated for 48 hours with either vehicle (0.1% DMSO [Sigma]) or 20 μM of etoposide (MilliporeSigma) dissolved in vehicle, followed by maintenance in their respective growth media until the indicated time points. Cells were lysed for RNA in QIAzol (QIAGEN) and stored at –80°C.

### Quantitative real-time polymerase chain reaction analysis.

Purification of RNA from QIAzol-lysed samples was performed with RNeasy Mini Columns (QIAGEN) with RNase-free DNase solution (QIAGEN) applied to degrade contaminating genomic DNA. RNA quantity was assessed with NanoDrop spectrophotometry (Thermo Fisher Scientific). cDNA synthesis was performed using Applied Biosystems High-Capacity cDNA Reverse Transcription Kit (Thermo Fisher Scientific). Transcript mRNA levels were determined by qRT-PCR on the ABI Prism 7900HT Real-Time System (Applied Biosystems, Thermo Fisher Scientific), using SYBR Green (QIAGEN). Mouse primer sequences were designed using Primer Express Software Version 3.0 (Applied Biosystems, Thermo Fisher Scientific) and are provided in Supplemental Table 2. Input RNA was normalized using *Actb* and *Tuba1a* as reference genes. For each sample (run in triplicate), the median cycle threshold (Ct) of each gene was normalized to the geometric mean of the median Ct of both reference genes. The ΔCt for each gene was used to calculate the relative mRNA expression changes for each sample. Genes with Ct values > 35 were considered not expressed.

### RNA-seq.

RNA-seq was performed on a HiSeq 4000 (Illumina), FASTQ files were mapped to the murine reference genome mm10, and analysis was performed as previously described (30, 84). Differentially expressed genes were calculated using DESeq2 (85), and GSEA was performed and visualized using the clusterProfiler and enrichplot packages (86). Genes included in the antiapoptotic gene set were selected from the BCL2 family (87), inhibitor of apoptotic proteins family (88), and other antiapoptotic gene analyses (89) and can be found in Supplemental Table 3. YAP/TAZ activity was assessed with the Cordenonsi Yap Conserved Signature (90) gene set obtained from the Molecular Signatures Database (https://www.gsea-msigdb.org/gsea/msigdb/index.jsp) and converted from human to mouse genes using the orthogene package (91) in R (v4.4.2).

### Multiplexed cytokine analysis.

Conditioned media were collected from control or senescent BMDM or BMSC cultures (treated as described above), filtered, and stored at –80°C until analysis. Serum was collected from untreated young (6-month) mice and old (24-month) *LysM-LOX-ATTAC* mice treated with either vehicle or AP. Samples were sent to Eve Technologies for analysis using the Mouse Cytokine/Chemokine 68-Plex Discovery Assay Array (MD68). Each specimen on each panel was run in singlet on the same analyzers, which is certified to perform high-complexity laboratory testing under the Clinical Laboratory Improvement Amendments (CLIA).

### Statistics.

Graphical data are shown as median ± minimum and maximum values (box plots) or mean ± SD (bar plots) unless otherwise specified. Sample sizes were determined based on previously conducted and published experiments (17, 41) in which statistically significant differences were observed among bone parameters or single-cell data in response to multiple interventions in our laboratory. Animal numbers per experiment are indicated in the figure legends, and all samples represent biological replicates. Data were examined for normality and distribution using the Shapiro-Wilk normality test. If the normality or equal variance assumptions for parametric analysis methods were not met, data were analyzed using nonparametric tests (e.g., Wilcoxon’s rank sum test, Mann-Whitney *U* test). For parametric tests, depending on the comparison, differences between groups were analyzed by unpaired *t* test (2 tailed) or 1- or 2-way ANOVA with pairwise multiple comparisons performed using the Tukey’s post hoc method, unless specified otherwise; the specific statistical test used for each comparison is detailed in each figure legend. Statistical analyses were performed using either GraphPad Prism (Version 9.0) or R version 4.0.2. A *P* value less than 0.05 was considered statistically significant.

### Study approval.

All animal studies were performed under protocols approved by the Mayo Clinic IACUC, and experiments were performed in accordance with Mayo Clinic IACUC guidelines.

### Data availability.

The scRNA-seq data were analyzed from the publicly available Tabula Muris dataset (36). The CyTOF data are available at Mendeley Data, V1, doi: 10.17632/tk466mks85.1. Bulk RNA-seq raw data have been deposited in the NCBI GEO and are available from GSE294362 (nonskeletal tissues from LysM-LOX-ATTAC mice) and GSE283633 (etoposide-treated BMSCs vs. macrophages; freshly isolated monocytes from young and old C57BL/6 mice). Values for all data points in graphs are reported in the Supporting Data Values file.

Code for reanalysis of the Tabula Muris Senis dataset and analysis of bulk RNA-seq data can be found at https://github.com/DoolittleLab/Doolittle-JCI-2026, commit ID cd9881b. CyTOF data were analyzed in software that does not require code (Cytobank).

## Author contributions

MLD and SK conceived the study. Experimental procedures were performed by MLD, MNF, JLR, MR, LS, JNF, and DGM. MLD and SK were responsible for data analysis. MLD wrote an initial draft of the manuscript. SK edited the manuscript. All authors reviewed the manuscript.

## Funding support

This work is the result of NIH funding, in whole or in part, and is subject to the NIH Public Access Policy. Through acceptance of this federal funding, the NIH has been given a right to make the work publicly available in PubMed Central.

- NIH grant P01 AG062413 (SK, JNF).
- NIH grant R01 AG076515 (SK, DGM).
- NIH grant R01 AG086085 (SK, DGM, MLD).
- NIH grant U54 AG079754 (SK).
- Hevolution HR-GRO-23-1199144-8 (SK, DGM).
- NIH grant R01 DK128552 (JNF).
- NIH grant R01 AG063707 (DGM).
- Mayo Clinic Edward C. Kendall Fellowship (MLD).

## Supplementary Material

Supplemental data

Supporting data values

## Version 1 01/27/2026

In-Press Preview

## Version 2 04/01/2026

Electronic publication
