# Supplementary material for: Aged murine bone marrow myeloid and mesenchymal cells develop unique senescence phenotypes
Source: J Clin Invest. 2026 Jan 27;136(7):e195772. doi: 10.1172/JCI195772 (PMC13038201; doi:10.1172/JCI195772)
Supplement: Supplemental data [file jci-136-195772-s165.pdf]

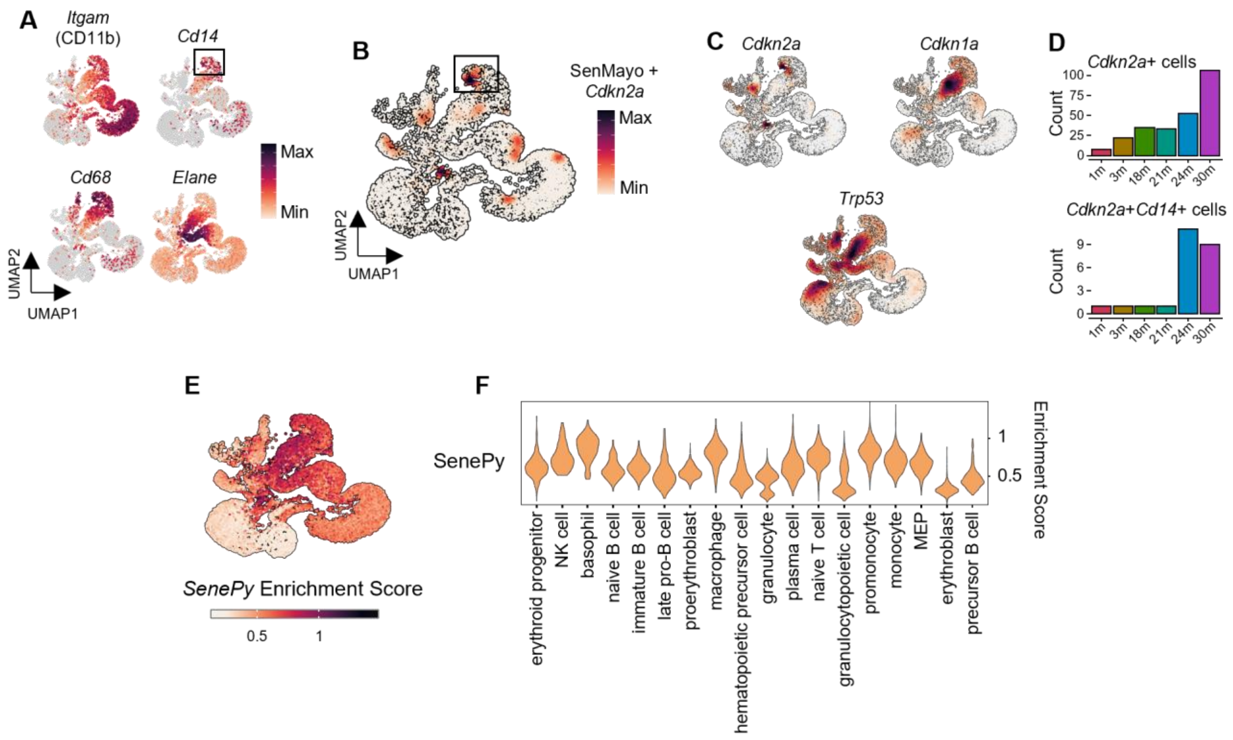

Supplementary Figure 1. Senescence marker expression within myeloid-lineage bone marrow cells. (**A-C**) Clustered bone marrow cell scRNA-seq data from the Tabula Muris Senis database, displaying only samples from 24-month (24m) and 30-month (30m) mouse cohorts, with UMAP plots demonstrating (**A**) individual myeloid gene expression, (**B**) cells jointly expressing both SenMayo and *Cdkn2a*, and (**C**) senescence-associated cyclin-dependent kinase inhibitor genes. N=8 male mice total (N=4 per age group). (**D**) Counts of *Cdkn2a*+ and *Cdkn2a*+*Cd14*+ bone marrow cells across the mouse lifespan. N=2-11 mice per age group. (**E, F**) Enrichment score using the *Senepy* (1) scoring system across bone marrow immune cell populations.

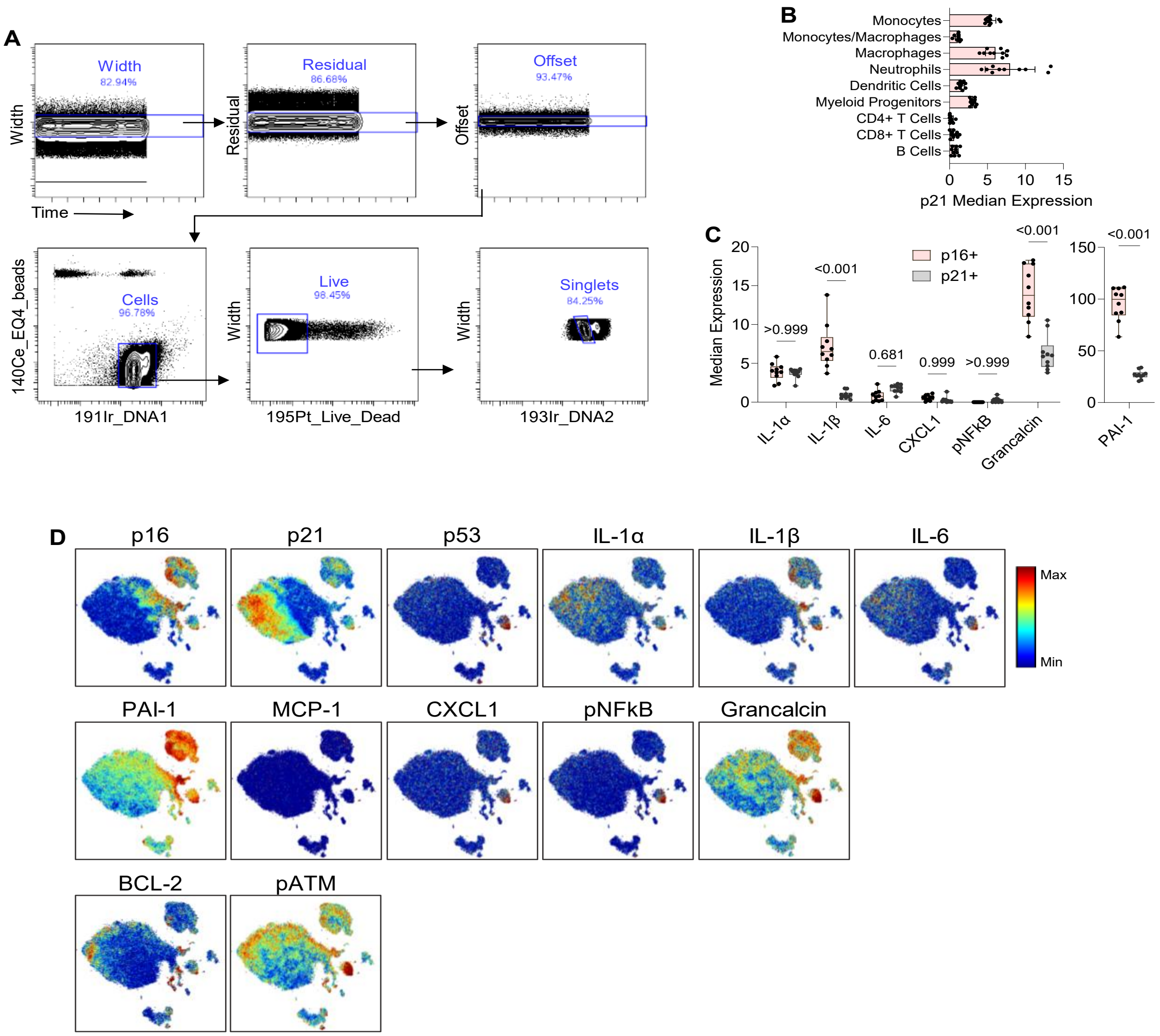

Supplementary Figure 2. CyTOF analysis of aged bone marrow immune cells. (**A**) Gating strategy for CyTOF sample pre-analysis processing. (**B**) p21 median expression levels across bone marrow immune clusters in 24- month-old wild-type C57BL/6N mice (corresponding to Figure 1E). N=10 24-month-old female mice. (**C**) SASP median expression levels between p16^{+} or p21^{+} bone marrow immune cells. Significance was determined by either unpaired t test or Mann Whitney test, as appropriate. (**D**) Expression plots of senescence-related proteins across all clusters (corresponding to Figure 1G).

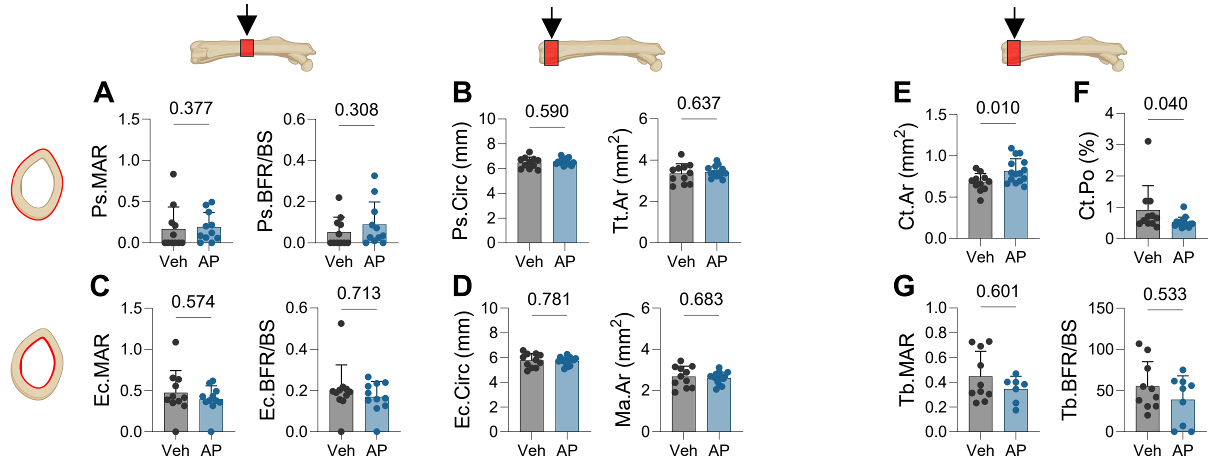

Supplementary Figure 3. Advanced skeletal phenotyping of *LysM-LOX-ATTAC* mice. Periosteal and endosteal analyses at the mid-shaft diaphysis by dynamic histomorphometry (**A, C**) and metaphysis by microCT (**B, D**). (**E, F**) Cortical area (Ct.Ar) and porosity (Ct.Po) at the femur metaphysis. (**G**) Dynamic histomorphometry values from the metaphyseal trabecular compartment. Ps = periosteal, Ec = endosteal, Tb = trabecular, MAR = mineral apposition rate, BFR/BS = bone formation rate/bone surface, Circ = circumference, Tt.Ar = total area, Ma.Ar = medullary area. N=11 per group; Significance was determined by either unpaired t test or Mann Whitney test, as appropriate.

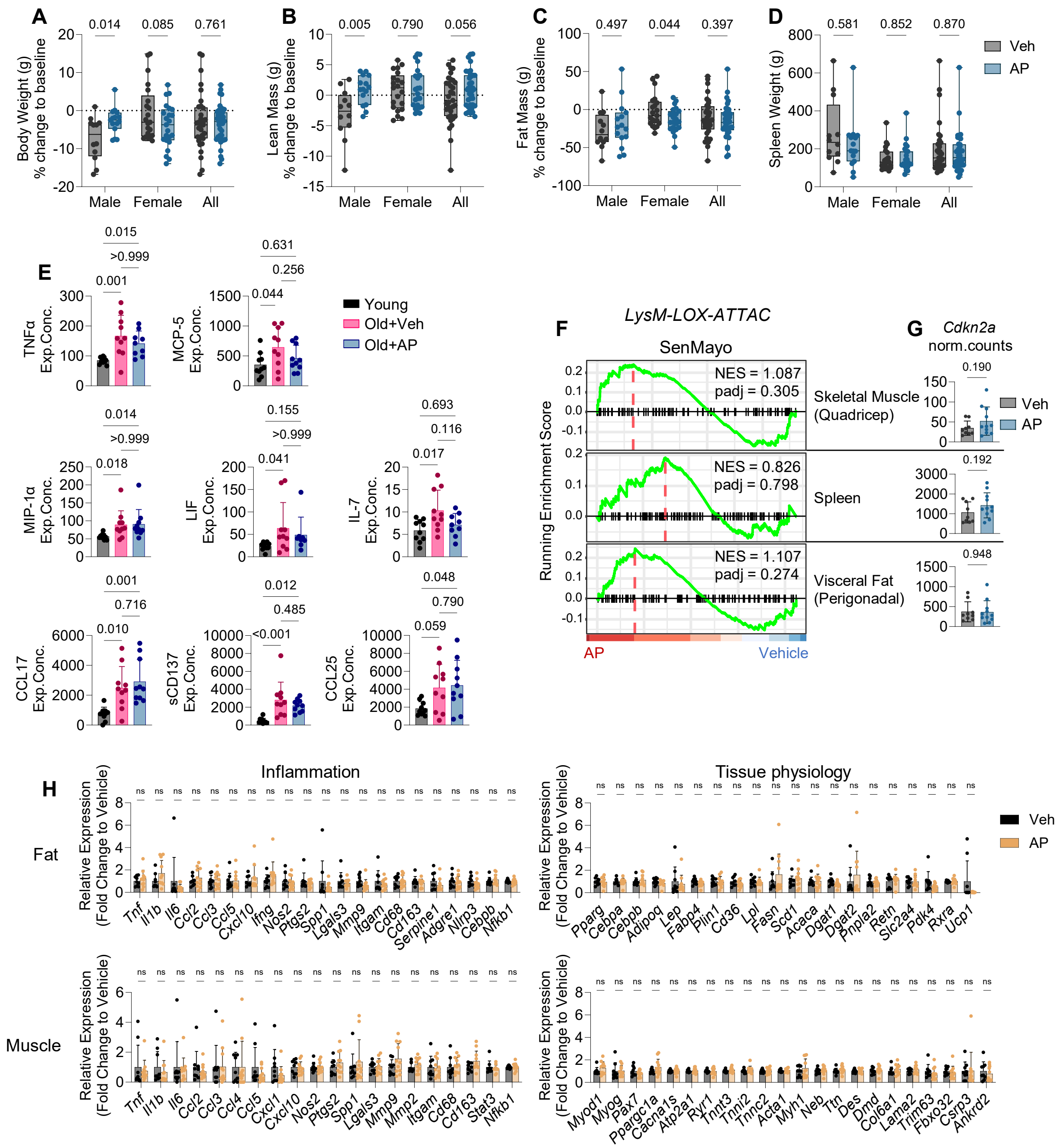

Supplementary Figure 4. Extraskeletal phenotyping in LysM-LOX-ATTAC mice. (**A-D**) Weight measurements of 24-month-old *LysM-LOX-ATTAC* mice, including body weight, lean mass, fat mass, and spleen weight at sacrifice, showing percent change from baseline to endpoint after 4 months of either vehicle or AP injections, stratified by sex. N=44 females (N=22 vehicle, N=22 AP) N=26 males (N=11 vehicle, N=15 AP). (**E**) Cytokine analysis of serum from *LysM-LOX-ATTAC* mice including untreated young mice (6-month) or old mice (24-month) treated with either vehicle or AP. N=10 per group, all males. (**F**) SenMayo GSEA and (**G**) *Cdkn2a* mRNA normalized counts in skeletal muscle (quadriceps), spleen, and visceral (perigonadal) fat tissue samples isolated from vehicle- or AP-treated *LysM-LOX-ATTAC* mice. N= 9 vehicle, N=11 AP, all male. Significance was determined by unpaired t-test or Mann Whitney test, as appropriate (**A-D, H**) or Kruskal-Wallis test with Dunn's multiple comparisons test (**E**).

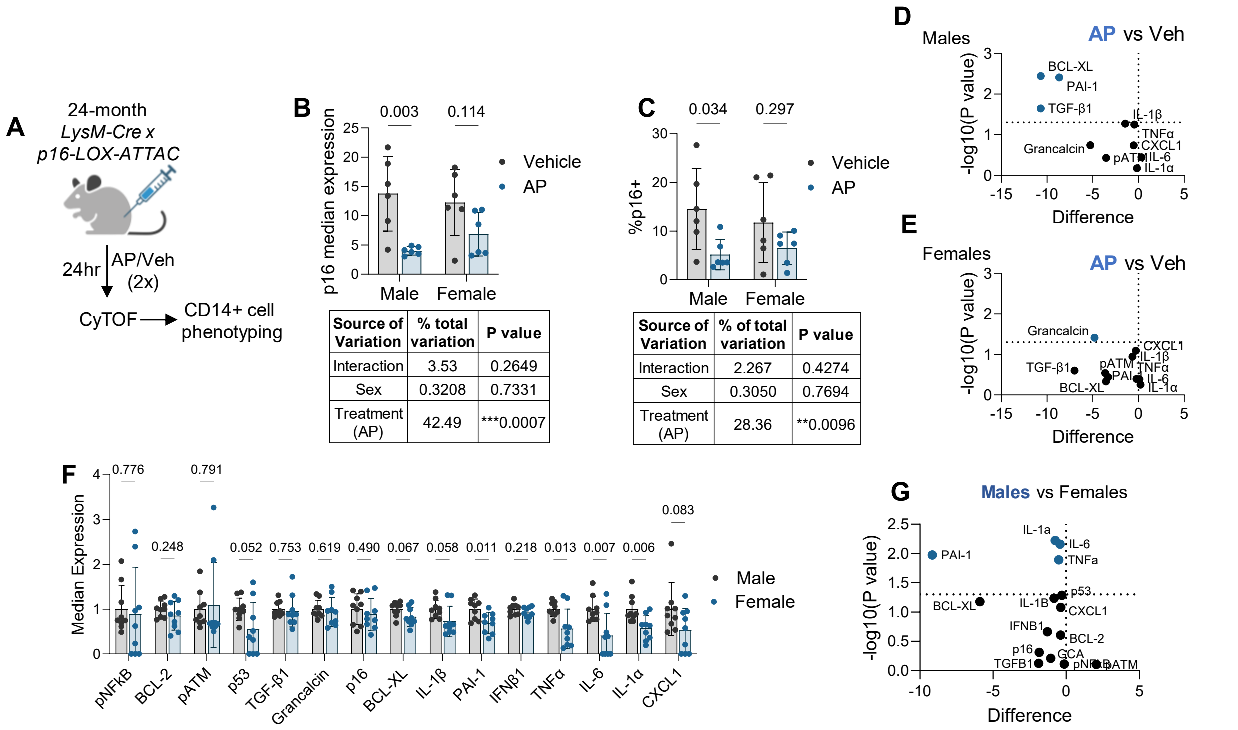

Source of Variation	% total variation	P value
Interaction	3.53	0.2649
Sex	0.3208	0.7331
Treatment (AP)	42.49	***0.0007

Source of Variation	% of total variation	P value
Interaction	2.267	0.4274
Sex	0.3050	0.7694
Treatment (AP)	28.36	**0.0096

Supplementary Figure 5. p16$^+$ myeloid cell clearance is more robust in males with a greater reduction in inflammation due to higher baseline inflammaging. (**A**) Schematic for CyTOF analysis of sex differences at 24 hours post-injection of either vehicle or AP in 24-month-old *LysM-LOX- ATTAC* mice. N=12 mice per treatment (6 male, 6 female). (**B, C**) Bar plots and 2-way ANOVA results of p16 median expression and %p16$^+$ cells in CD14$^+$ bone marrow cells at 24 hours after vehicle or AP treatment. (**D, E**) Volcano plots of SASP marker differential expression between AP- versus vehicle-treated mice in males or females. (**F**) Bar plots and (**G**) volcano plot of SASP marker differential expression between wild-type male or female mice. N=9 mice per sex. Significance was determined by 2-way ANOVA with Tukey multiple comparisons test (**B, C**) and by either unpaired t tests or Mann Whitney tests, as appropriate (**D-G**).

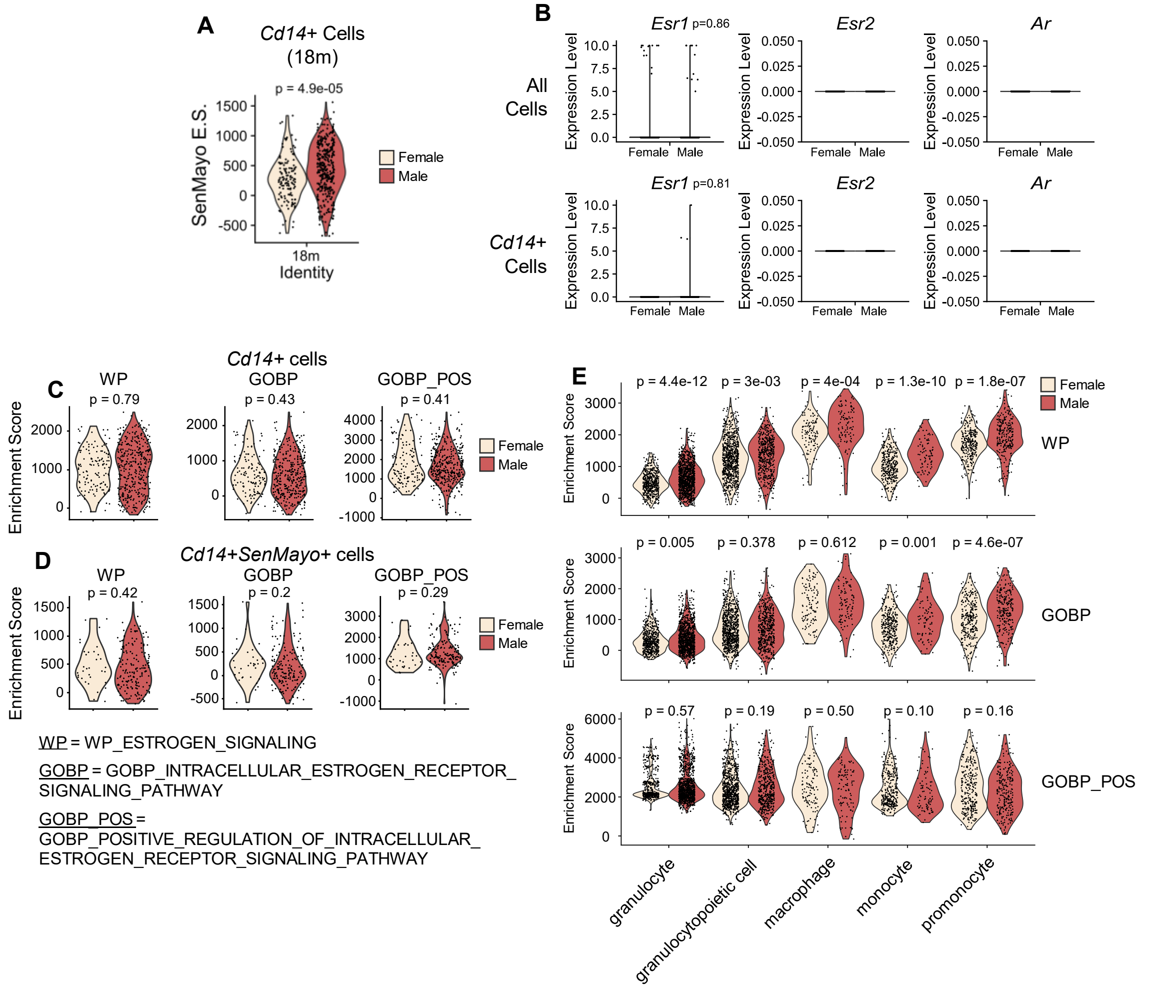

Supplementary Figure 6. Sex hormone signaling signatures do not correlate with sex-dependent senescence burden in aged myeloid cells. (**A**) SenMayo enrichment score in *Cd14*+ bone marrow cells from 18-month male and female mice (Tabula Muris Senis dataset). (**B**) mRNA expression of genes encoding sex hormone receptors (*Esr1* [ERα], *Esr2* [ERβ], and *Ar* [androgen receptor]). (**C-E**) Estrogen signaling gene set enrichment score across three separate estrogen signaling gene sets in (**C**) *Cd14*+, (**D**) *Cd14*+SenMayo+, and (**E**) myeloid-lineage bone marrow immune cells. N=2 males, N=4 females. Significance was determined using the Wilcoxon rank-sum test (**A-D**), and Kruskal-Wallis test (**E**).

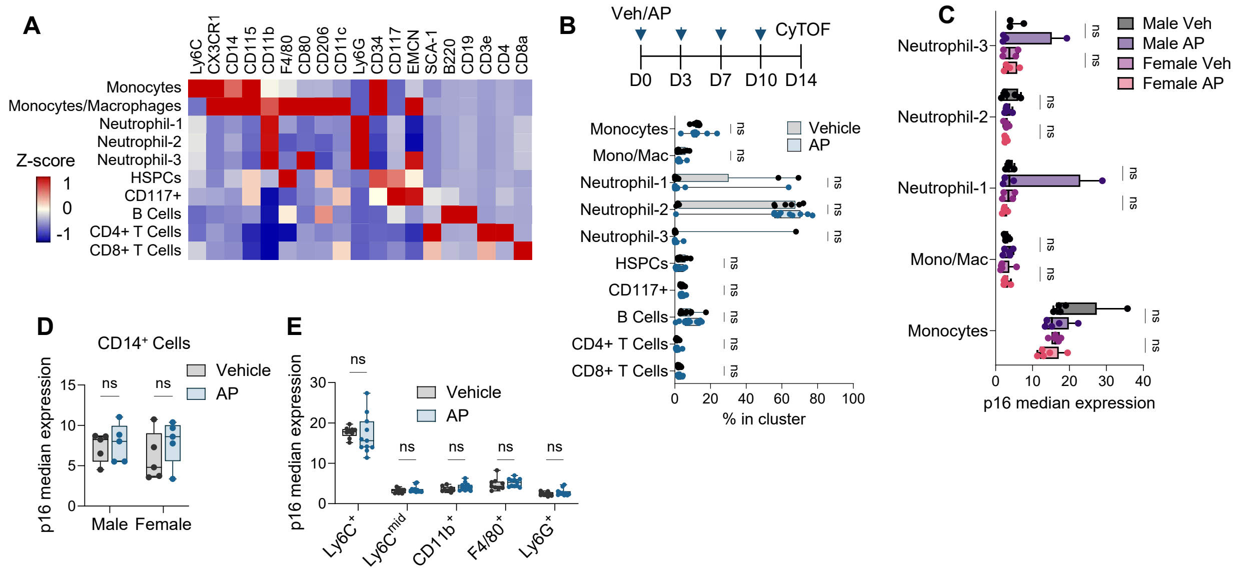

Supplementary Figure 7. Supporting data for two-week *LysM-LOX-ATTAC* CyTOF analyses. (**A**) Heatmap showing median expression of identifying markers across bone marrow immune populations from treated *LysM- LOX-ATTAC* mice analyzed by CyTOF. (**B**) Percentage of cells in each cell cluster from mice treated with either vehicle or AP. N=9 vehicle (4 males, 5 females), N=11 AP (5 males, 6 females). Significance was determined by either unpaired t-test or Mann Whitney test, as appropriate. (**C**) Sex-stratified analyses of p16 median expression between vehicle- and AP-treated mice. Significance was determined by 2-way ANOVA with Tukey multiple comparisons test. (**D-E**) p16 median expression in (**D**) $CD14^{+}$ cells and (**E**) common myeloid-lineage cell types from mice treated with either vehicle or AP for two weeks. Significance was determined by either unpaired t-test or Mann Whitney test, as appropriate.

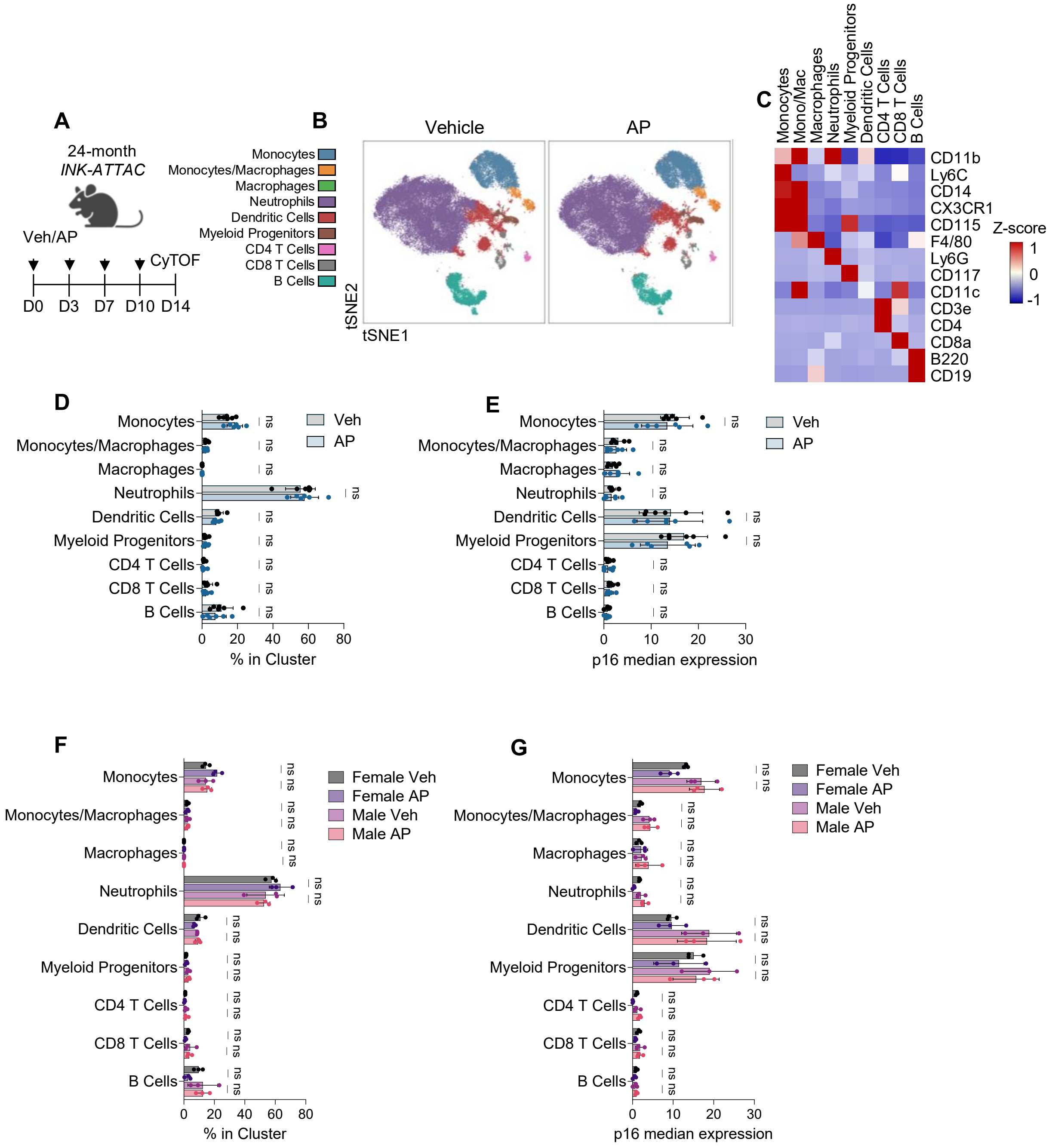

Supplementary Figure 8. Global senolytic therapy does not lead to stable removal of p16⁺ bone marrow immune cells. (**A**) Schematic showing global clearance of p16⁺ cells in *INK-ATTAC* mice with 2-week treatment of either vehicle or AP. N=6 mice per treatment (3 males, 3 females). (**B**) tSNE visualization of clustered bone marrow cells from treated *INK-ATTAC* mice. (**C**) Heatmap representation of median expression levels of identification markers for bone marrow immune cells. (**D, E**) Cell percentages and p16 median expression levels in each bone marrow immune cell population between vehicle- and AP-treated mice. Significance was determined by unpaired t-tests. (**F, G**) Cell percentages and p16 median expression levels in each bone marrow immune cell population between vehicle- and AP-treated mice, stratified by sex. Significance was determined by 2-way ANOVA with Tukey multiple comparisons test.

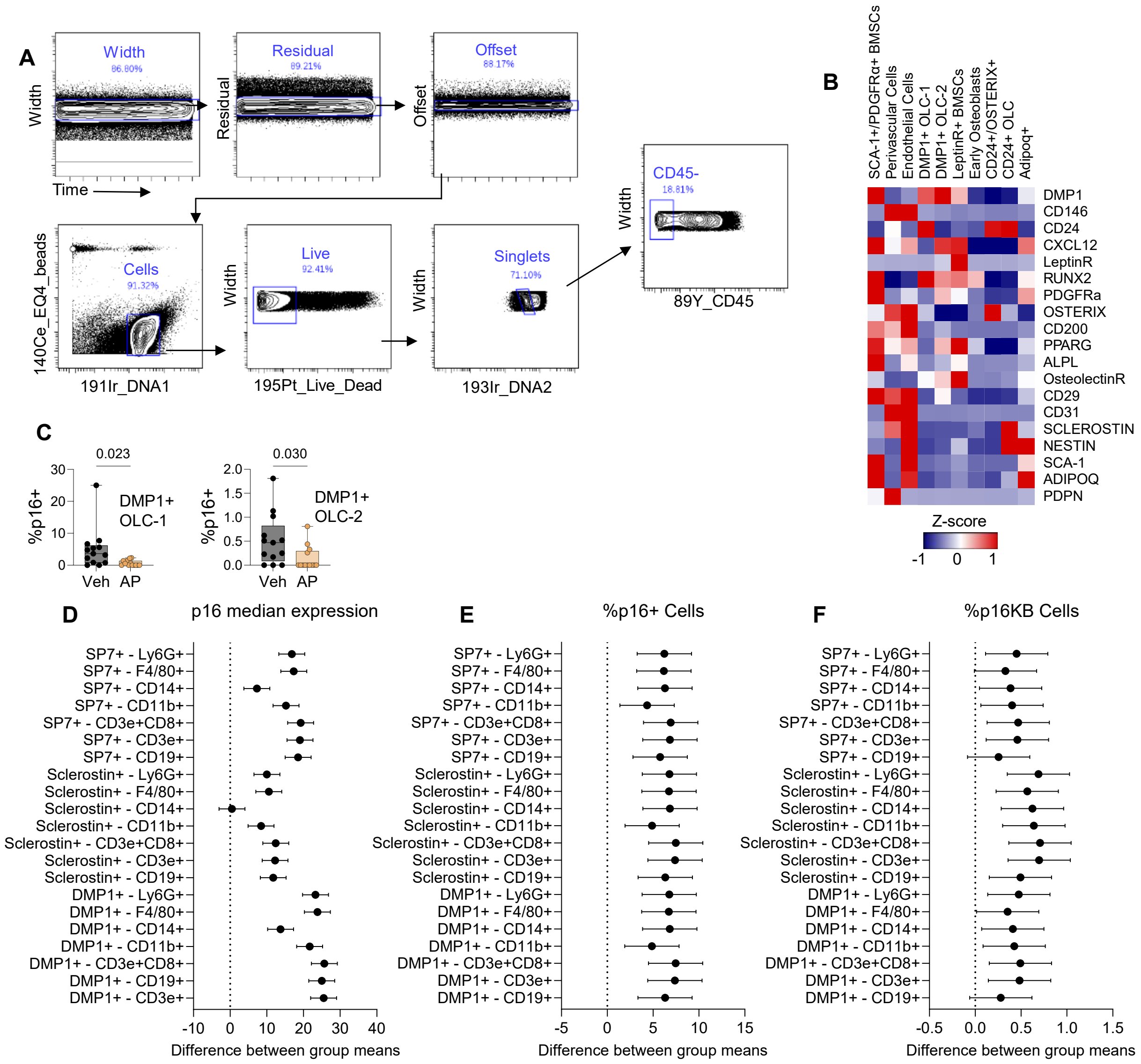

Supplementary Figure 9: *DMP-LOX-ATTAC* CyTOF supporting data and statistical comparisons of senescence parameters between mesenchymal cells and immune cells. (**A**) Gating strategy for the analysis of mesenchymal-lineage bone cells by CyTOF. (**B**) Heatmap showing median expression of identifying markers across mesenchymal-lineage bone cell populations from treated *DMP-LOX-ATTAC* mice analyzed by CyTOF. (**C**) %p16+ cells in DMP1+ osteolineage (OLC) clusters. N=13 per group. Significance was determined by unpaired t test. (**D-F**) One-way ANOVA results on corresponding data from Figure 5I-K showing differences in group means of osteolineage cells versus various immune cell types of (**D**) p16 median expression, (**E**) %p16^{+} cells, and (**F**) %p16KB cells between mesenchymal and immune populations. Error bars are 95% confidence intervals. N=18 mice total (9 males, 9 females).

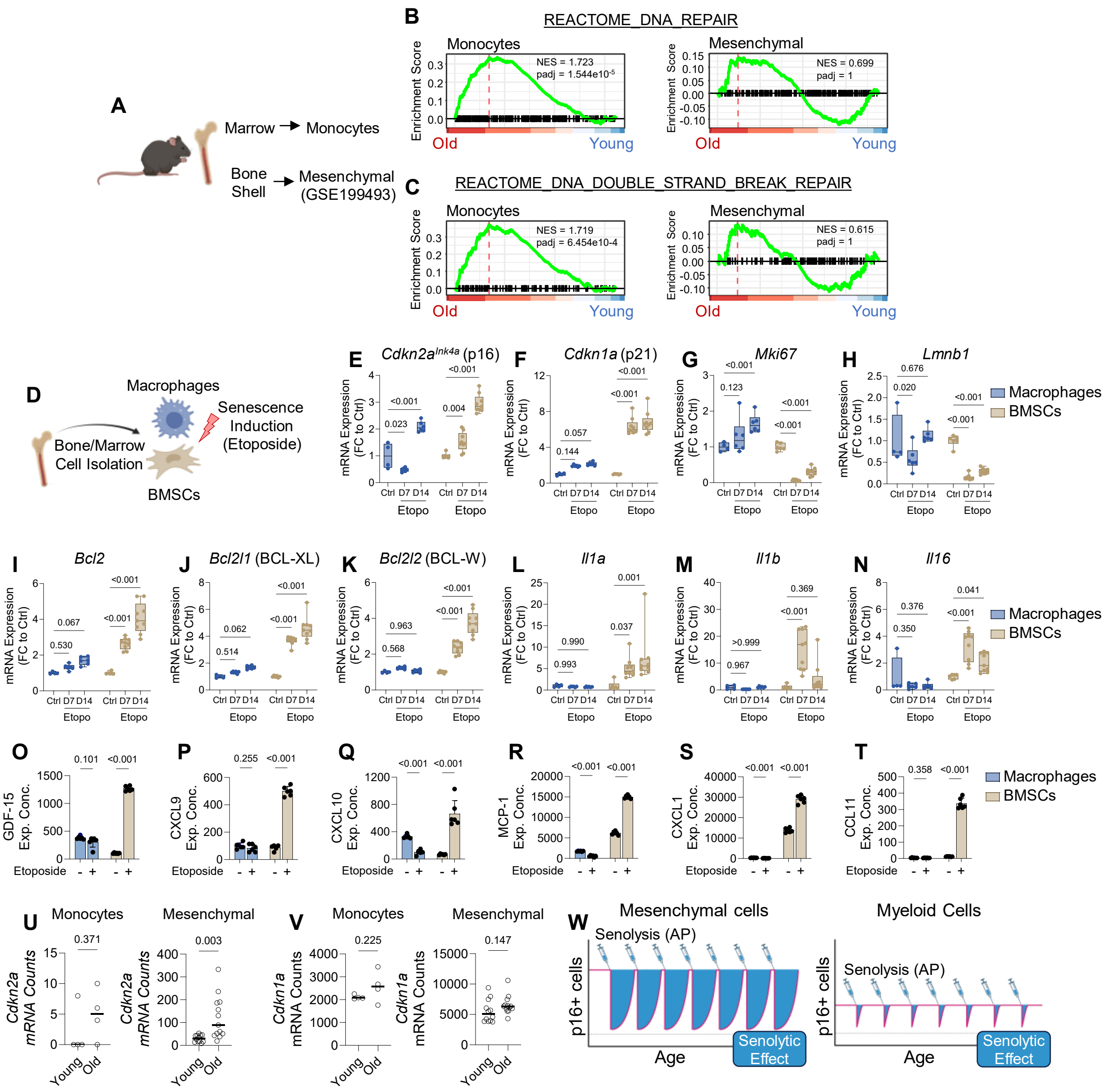

Supplementary Figure 10: Senescence markers are expressed in unique patterns between mesenchymal- and myeloid-lineage cells. (**A**) Freshly-isolated bone marrow monocytes from young (6-month) and old (24-month) mice (N=4 per age) and age-matched mesenchymal-enriched diaphysis bone samples from previous work (N=12-13 per age) (2) (GSE199493). (**B, C**) GSEA plots of DNA repair and DNA double strand break repair gene sets in monocyte and mesenchymal samples. (**D-N**) qPCR results from senescence induction experiments, demonstrating relative mRNA levels of senescence-associated genes. N=4-9 per time point. Significance was determined by 2-way ANOVA with Tukey multiple comparisons test. (**O-T**) Quantification of cytokine array from conditioned media from control or D14 etoposide-treated macrophages or BMSCs. N=6 per group. Significance was determined by multiple t-tests with Holm-Sidak correction. (**U**) *Cdkn2a* and (**V**) *Cdkn1a* mRNA counts from freshly-isolated CD14+ monocytes and mesenchymal-enriched bone diaphysis samples (GSE199493); line at median. Significance was determined by unpaired t-test (**W**) Summarized schematic of findings, indicating a larger senolytic effect from targeting mesenchymal p16$^+$ cells (rather than myeloid p16$^+$ cells) due to their higher expression of senescence markers and longer period of removal after senolysis.

Supplementary Table 1. CyTOF Antibody Panels.

Immune Senescence Phenotyping (Figure 1)

Marker	Metal	Vendor	Cat#
CD45	89Y	Standard BioTools	3089005B
CD4	145Nd	Biolegend	100506
CD34	163Dy	Thermo Fisher	MA5-17826
CD11c	142Nd	Biolegend	117310
CD8a	168Er	Biolegend	100702
CD117	173Yb	Standard BioTools	3173004B
CD3e	152Sm	Biolegend	100302
CD19	166Er	Biolegend	115502
pNFkB	110Cd	Cell Signaling	3033
BCL-2	112Cd	Biolegend	633502
pATM	114Cd	Abcam	ab217838
p53	116Cd	Abcam	ab252388
MCP-1	149Sm	Thermo Fisher	MA5-17040
p16	155Gd	Abcam	ab232402
IL-1B	159Tb	Cell Signaling	31202
PAI-1	160Gd	Abcam	ab125687
IL-6	167Er	Standard BioTools	3167003B
SCA-1	169Tm	Standard BioTools	3169015B
IL-1a	171Yb	Biolegend	503202
CXCL1	175Lu	R&D Systems	MAB453-500
p21	176Yb	Santa Cruz	sc-6246
CD11b	111Cd	Biolegend	101201
CCR2	156Gd	Novus Biologicals	MAB55381-100
F4/80	113Cd	Biolegend	123101
Ly6C	141Pr	Biolegend	128039
CD28	143Nd	Biolegend	102101
Grancalcin	146Nd	Bosterbio	A01237
CTSK	147Sm	Abcam	ab37259
CD80	148Nd	Biolegend	104735
Granzyme K	150Nd	LSBio	LS-C176975-100
CD206	151Eu	Biolegend	141702
B220	153Eu	Biolegend	103249
CD265	154Sm	ThermoFisher	14-6612-82
Catalase	158Gd	Abcam	ab223793
CX3CR1	161Dy	Biolegend	149002
EMCN	164Dy	ThermoFisher	14-5851-82
CD14	165Ho	Biolegend	123321
Ly6G	170Er	Biolegend	127637
CD115	174Yb	Biolegend	135502

LysM-LOX-ATTAC CyTOF (Figure 3)

**Marker**	**Metal**	**Vendor**	**Cat#**
CD45	89Y	Standard BioTools	3089005B
CD4	145Nd	Biolegend	100506
CD34	163Dy	Thermo Fisher	MA5-17826
CD11c	142Nd	Biolegend	117310
CD8a	168Er	Biolegend	100702
CD117	173Yb	Standard BioTools	3173004B
CD3e	152Sm	Biolegend	100302
CD19	166Er	Biolegend	115502
Ki67	106Cd	Standard BioTools	3168007B
pNFkB	110Cd	Cell Signaling	3033
BCL-2	112Cd	Biolegend	633502
pATM	114Cd	Abcam	ab217838
p53	116Cd	Abcam	ab252388
MCP-1	149Sm	Thermo Fisher	MA5-17040
p16	155Gd	Abcam	ab232402
IL-1B	159Tb	Cell Signaling	31202
PAI-1	160Gd	Abcam	ab125687
IL-6	167Er	Standard BioTools	3167003B
SCA-1	169Tm	Standard BioTools	3169015B
IL-1a	171Yb	Biolegend	503202
CXCL1	175Lu	R&D Systems	MAB453-500
p21	176Yb	Santa Cruz	sc-6246
CD11b	111Cd	Biolegend	101201
CCR2	156Gd	Novus Biologicals	MAB55381-100
F4/80	113Cd	Biolegend	123101
Ly6C	141Pr	Biolegend	128039
CD28	143Nd	Biolegend	102101
Grancalcin	146Nd	Bosterbio	A01237
CTSK	147Sm	Abcam	ab37259
CD80	148Nd	Biolegend	104735
Granzyme K	150Nd	LSBio	LS-C176975-100
CD206	151Eu	Biolegend	141702
B220	153Eu	Biolegend	103249
CD265	154Sm	ThermoFisher	14-6612-82
Catalase	158Gd	Abcam	ab223793
CX3CR1	161Dy	Biolegend	149002
EMCN	164Dy	ThermoFisher	14-5851-82
CD14	165Ho	Biolegend	123321
Ly6G	170Er	Biolegend	127637
CD115	174Yb	Biolegend	135502
TGF-B1	144Nd	Novus Biologicals	NBP2-74495

DMP-LOX-ATTAC (Figure 3)

Marker	Metal	Vendor	Cat#
CD45	89Y	Standard BioTools	3089005B
CD140a/PDGFRα	148Nd	BioLegend	135902
Ki67	106Cd	Biolegend	350523
pNFkB	110Cd	Cell Signaling	3033
OCN	111Cd	Santa Cruz	sc-376835
BCL-2	112Cd	Biolegend	633502
pATM	114Cd	Abcam	ab217838
p53	116Cd	Abcam	ab252388
CD146	141Pr	Standard BioTools	3141016B
CD24	142Nd	Biolegend	101802
DMP-1	143Nd	ThermoFisher	PA5-47621
CXCL12	144Nd	R&D Systems	MAB350-100
CENP-B	145Nd	Santa Cruz	sc-376283
LeptinR	146Nd	R&D Systems	AF497
Runx2	147Sm	Abcam	ab76956
MCP-1	149Sm	Thermo Fisher	MA5-17040
Osterix	151Eu	Invitrogen	PA5-40411
CD200	152Sm	Biolegend	123802
PPARg	153Eu	Invitrogen	PA3-821A
ALPL	154Sm	R&D Systems	af2910
p16	155Gd	Abcam	ab232402
Itga11	156Gd	Abcam	ab198826
CD29	158Gd	Biolegend	102235
IL-1B	159Tb	Cell Signaling	31202
PAI-1	160Gd	Abcam	ab125687
TNFa	162Dy	Standard BioTools	3162002B
FLAG	164Dy	Biolegend	637301
CD31	165Ho	Standard BioTools	3165013B
Sclerostin	166Er	Abcam	ab63097
IL-6	167Er	Standard BioTools	3167003B
Nestin	168Er	Biolegend	655102
SCA-1	169Tm	Standard BioTools	3169015B
Adiponectin	170Er	Invitrogen	PA1-84881
IL-1a	171Yb	Biolegend	503202
yH2A-X	173Yb	BD Biosciences	560443
PDPN	174Yb	BioLegend	127401
CXCL1	175Lu	R&D Systems	MAB453-500
p21	176Yb	Santa Cruz	sc-6246

Mesenchymal vs Immune Senescence Phenotyping – Immune Panel (Figure 4)

**Marker**	**Metal**	**Vendor**	**Cat#**
CD45	89Y	Standard BioTools	3089005B
CD34	163Dy	Thermo Fisher	MA5-17826
CD8a	168Er	Biolegend	100702
CD11c	209Bi	Biolegend	117310
CD3e	152Sm	Biolegend	100302
CD19	166Er	Biolegend	115502
Ki67	106Cd	Standard BioTools	3168007B
pNFkB	110Cd	Cell Signaling	3033
pATM	114Cd	Abcam	ab217838
p53	116Cd	Abcam	ab252388
p16	155Gd	Abcam	ab232402
TNFa	162Dy	Standard BioTools	3162002B
IL-6	167Er	Standard BioTools	3167003B
CXCL1	175Lu	R&D Systems	MAB453-500
p21	176Yb	Santa Cruz	sc-6246
CD11b	111Cd	Biolegend	101201
CD28	143Nd	Biolegend	102101
Grancalcin	146Nd	Bosterbio	A01237
CD80	148Nd	Biolegend	104735
CD206	151Eu	Biolegend	141702
Ly6G	170Er	Biolegend	127637
CD115	174Yb	Biolegend	135502
BCL-2	112Cd	Biolegend	633502
IL-1B	159Tb	Cell Signaling	31202
SCA-1	169Tm	Standard BioTools	3169015B
IL-1a	171Yb	Biolegend	503202
TGF-B1	144Nd	Novus Biologicals	NBP2-74495
Survivin	145Nd	ThermoFisher	MA5-15077
LaminB1	150Nd	Abcam	ab239399
BCL-XL	156Gd	Abcam	ab32370
PAI-1	160Gd	Abcam	ab125687
IFNB1	161Dy	Biolegend	508102
p38-MAPK	164Dy	ThermoFisher	MA5-15182
Ly6C	198Pt	Biolegend	128039
F4/80	113Cd	Biolegend	123101
CD4	141Pr	Biolegend	100506
CXCR4	142Nd	Biolegend	146502
CTSK	147Sm	Abcam	ab37259
CD117	153Eu	Standard BioTools	3173004B
CD14	154Sm	Biolegend	123321
CD62L	158Gd	Biolegend	104402
CCR5	165Ho	BD Biosciences	559921

Mes vs Imm Senescence Phenotyping – Mesenchymal Panel (Figure 4)

Marker	Metal	Vendor	Cat#
CD45	89Y	Standard BioTools	3089005B
CD31	163Dy	Standard BioTools	3165013B
TNFa	162Dy	Standard BioTools	3162002B
IL-6	167Er	Standard BioTools	3167003B
pNFkB	110Cd	Cell Signaling	3033
BCL-2	112Cd	Biolegend	633502
pATM	114Cd	Abcam	ab217838
p53	116Cd	Abcam	ab252388
Grancalcin	146Nd	Bosterbio	A01237
p16	155Gd	Abcam	ab232402
PAI-1	160Gd	Abcam	ab125687
CXCL1	175Lu	R&D Systems	MAB453-500
p21	176Yb	Santa Cruz	sc-6246
Ki67	106Cd	Standard BioTools	3168007B
TGF-B1	144Nd	Novus Biologicals	NBP2-74495
Survivin	145Nd	ThermoFisher	MA5-15077
LaminB1	150Nd	Abcam	ab239399
IFNB1	161Dy	Biolegend	508102
p38-MAPK	164Dy	ThermoFisher	MA5-15182
CD146	141Pr	Standard BioTools	3141016B
LeptinR	113Cd	R&D Systems	AF497
Nestin	168Er	Biolegend	655102
SCA-1	169Tm	Standard BioTools	3169015B
CD24	142Nd	Biolegend	101802
CD140a/PDGFRα	148Nd	BioLegend	135902
CXCL12	174Yb	R&D Systems	MAB350-100
CD200	152Sm	Biolegend	123802
CD29	158Gd	Biolegend	102235
TRAF3	165Ho	Santa Cruz	sc-6933
CCL5	172Yb	Santa Cruz	sc-373984
Runx2	147Sm	Abcam	ab76956
Osterix	151Eu	Invitrogen	PA5-40411
ALPL	154Sm	R&D Systems	af2910
OCN	111Cd	Santa Cruz	sc-376835
DMP-1	143Nd	ThermoFisher	PA5-47621
Sclerostin	166Er	Abcam	ab63097
PPARg	153Eu	Invitrogen	PA3-821A
Adiponectin	170Er	Invitrogen	PA1-84881
IL-1a	171Yb	Biolegend	503202
IL-1B	159Tb	Cell Signaling	31202
BCL-XL	156Gd	Abcam	ab32370

Supplementary Table 2. qPCR Primer Sequences.

Gene	Forward Primer sequence (5'-3')	Reverse Primer Sequence (5'-3')
Cdkn2a^{INK4a}	GAACTCTTTCGGTCGTACCC	AGTTCGAATCTGCACCGTAGT
Cdkn1a	GAACATCTCAGGGCCGAAAA	TGCGCTTGGAGTGATAGAAATC
Bcl2	CGGGAGATCGTGATGAAGTACA	ATCTCCAGCATCCCACTCGTA
Bcl2l1	AGGCCCCAGAAGAAACTGAAG	AGGATGGGTTGCCATTGATG
Bcl2l2	CCCTGTGTGCTGAGAGTGTCA	CCATCCAATCCTGCACTTGTC
Laminb1	TCTCCTCACAGCTCTCTAAC	CTCCAATTCCTGGATCCTTTC
Il1a	AAGAGACCATCCAACCCAGATC	CCTGACGAGCTTCATCAGTTTG
Il1b	TCAGGCAGGCAGTATCACTCA	CACGGGAAAGACACAGGTAGCT
Il6	ACCACGGCCTTCCCTACTTC	TTGGGAGTGGTATCCTCTGTGA
Mki67	AGACTGCCTCCCAGGAGACA	GGCCCCGAGATGTAGATTTCT
Actb	AATCGTGCGTGACATCAAAGAG	GCCATCTCCTGCTCGAAGTC
Tuba1a	GGTTCCCAAAGATGTCAATGCT	CAAACTGGATGGTACGCTTGGT

Supplementary Table 3. Anti-Apoptotic Gene Set.

Term	Gene
ANTI_APOPTOSIS	Bcl2
ANTI_APOPTOSIS	Bcl2l1
ANTI_APOPTOSIS	Bcl2l2
ANTI_APOPTOSIS	Mcl1
ANTI_APOPTOSIS	Cflar
ANTI_APOPTOSIS	Gadd45a
ANTI_APOPTOSIS	Naip1
ANTI_APOPTOSIS	Naip2
ANTI_APOPTOSIS	Naip3
ANTI_APOPTOSIS	Naip4
ANTI_APOPTOSIS	Naip5
ANTI_APOPTOSIS	Naip6
ANTI_APOPTOSIS	Birc2
ANTI_APOPTOSIS	Birc3
ANTI_APOPTOSIS	Xiap
ANTI_APOPTOSIS	Birc6
ANTI_APOPTOSIS	Birc7

Supplementary Table 4. Upstream Regulator Analysis results of RNA-seq data from etoposide-treated BMSCs and macrophages.

Cell Type	Upstream Regulator	Molecule Type	Predicted Activation State	Activation z-score	p-value of overlap
Macrophages	daidzein	chemical drug	Activated	2.607	5.04E-07
Macrophages	THRB	ligand-dependent nuclear receptor	Activated	2.425	3.79E-03
Macrophages	PKD1	ion channel	Activated	2.236	7.27E-03
Macrophages	SU6656	chemical toxicant	Activated	2.236	8.95E-07
Macrophages	trans-hydroxytamoxifen	chemical drug	Activated	2.236	1.82E-03
Macrophages	COL18A1	other	Activated	2.219	6.98E-05
Macrophages	CFTR	ion channel	Activated	2.219	7.52E-07
Macrophages	N-Ac-Leu-Leu-norleucinal	chemical - protease inhibitor	Activated	2.201	4.44E-05
Macrophages	PSMB11	peptidase	Activated	2.121	1.53E-07
Macrophages	KLF3	transcription regulator	Activated	2.121	3.64E-04
Macrophages	U0126	chemical drug	Activated	2.104	4.36E-08
Macrophages	SHC1	other	Activated	2.085	2.87E-05
Macrophages	YY1	transcription regulator	Activated	2.008	2.62E-08
Macrophages	PDCD10	other	Activated	2	1.13E-06
Macrophages	CDCA8	other	Activated	2	6.26E-04
Macrophages	LAPTM5	other	Activated	2	1.98E-04
Macrophages	SSTR2	G-protein coupled receptor	Activated	2	1.39E-06
Macrophages	TWF1	kinase	Activated	2	4.68E-04
Macrophages	baicalein	chemical drug	Activated	2	8.55E-04
Macrophages	OLFM4-RET	fusion gene/product	Inhibited	-2	8.92E-04
Macrophages	COP1	enzyme	Inhibited	-2	6.26E-04
Macrophages	NANOG	transcription regulator	Inhibited	-2	1.03E-02
Macrophages	CD83	transmembrane receptor	Inhibited	-2	1.62E-05
Macrophages	APEX1	enzyme	Inhibited	-2	2.08E-05
Macrophages	CREB (family)	group	Inhibited	-2.069	5.02E-04
Macrophages	pirinixic acid	chemical toxicant	Inhibited	-2.079	2.60E-06
Macrophages	RAS (family)	group	Inhibited	-2.097	4.70E-06
Macrophages	F2	peptidase	Inhibited	-2.098	1.39E-07
Macrophages	lipopolysaccharide	chemical drug	Inhibited	-2.1	1.45E-15
Macrophages	INSULIN (family)	group	Inhibited	-2.126	1.95E-05
Macrophages	IL5	cytokine	Inhibited	-2.157	1.23E-05
Macrophages	IL7	cytokine	Inhibited	-2.169	8.70E-05
Macrophages	quinolinic acid	chemical - endogenous	Inhibited	-2.174	3.37E-06

		mammalian			
Macrophages	MAPK3	kinase	Inhibited	-2.176	1.60E-04
Macrophages	bicuculline	chemical - endogenous non-mammalian	Inhibited	-2.183	1.03E-05
Macrophages	TRIM28	transcription regulator	Inhibited	-2.186	1.31E-03
Macrophages	5-azacytidine	chemical drug	Inhibited	-2.195	3.31E-03
Macrophages	CD38	enzyme	Inhibited	-2.2	1.53E-03
Macrophages	SPHK1	kinase	Inhibited	-2.202	8.68E-06
Macrophages	nitric oxide	chemical - endogenous mammalian	Inhibited	-2.202	1.62E-04
Macrophages	CASR	G-protein coupled receptor	Inhibited	-2.219	5.23E-04
Macrophages	GNAQ	enzyme	Inhibited	-2.226	1.40E-05
Macrophages	CREBBP	transcription regulator	Inhibited	-2.233	9.79E-07
Macrophages	isobutylmethylxanthine	chemical toxicant	Inhibited	-2.236	1.13E-05
Macrophages	TICAM1	other	Inhibited	-2.388	3.34E-09
Macrophages	tetradecanoylphorbol acetate	chemical drug	Inhibited	-2.457	4.37E-12
Macrophages	ESR2	ligand-dependent nuclear receptor	Inhibited	-2.458	1.40E-05
Macrophages	CG (complex)	complex	Inhibited	-2.525	2.54E-09
Macrophages	IGF1	growth factor	Inhibited	-2.648	1.89E-06
Macrophages	IFNG	cytokine	Inhibited	-3.047	2.11E-13
Macrophages	PDGF-BB (complex)	complex	Inhibited	-3.091	2.10E-09
BMSCs	TP53	transcription regulator	Activated	6.96	1.65E-41
BMSCs	NUPR1	transcription regulator	Activated	6.78	1.56E-17
BMSCs	IFNG	cytokine	Activated	6.683	2.1E-34
BMSCs	let-7 (includes others)	microRNA	Activated	6.655	3.04E-15
BMSCs	IRF3	transcription regulator	Activated	6.413	2.56E-18
BMSCs	IFN BETA (family)	group	Activated	6.368	3.36E-23
BMSCs	ZBTB10	transcription regulator	Activated	6.161	2.05E-21
BMSCs	STING1	ion channel	Activated	6.122	7.37E-19
BMSCs	IL1B	cytokine	Activated	5.796	1.72E-26
BMSCs	IRF7	transcription regulator	Activated	5.768	8.95E-12
BMSCs	STAT1	transcription regulator	Activated	5.741	4.95E-23
BMSCs	IFNA2	cytokine	Activated	5.658	1.02E-09
BMSCs	PAFAH1B1	enzyme	Activated	5.591	1.75E-19
BMSCs	IFNB1	cytokine	Activated	5.587	1.79E-23

BMSCs	IFNAR (family)	group	Activated	5.421	1.83E-14
BMSCs	CDKN2A	enzyme	Activated	5.195	2.32E-18
BMSCs	IFNAR1	transmembrane receptor	Activated	5.084	1.03E-12
BMSCs	CHUK	kinase	Activated	5.006	4.44E-16
BMSCs	TNF	cytokine	Activated	4.957	1.56E-32
BMSCs	CDKN1A	kinase	Activated	4.853	1.72E-43
BMSCs	TLR4	transmembrane receptor	Activated	4.754	6.36E-12
BMSCs	MAGI1	enzyme	Activated	4.743	1.07E-16
BMSCs	SMARCB1	transcription regulator	Activated	4.7	1.18E-16
BMSCs	SENP3	peptidase	Activated	4.679	4.26E-15
BMSCs	INTERFERON ALPHA (family)	group	Activated	4.665	2.83E-23
BMSCs	IL1A	cytokine	Activated	4.592	1.95E-13
BMSCs	TLR3	transmembrane receptor	Activated	4.553	3.13E-11
BMSCs	IFNA4	cytokine	Activated	4.405	2.35E-13
BMSCs	IMMUNOGLOBULIN (complex)	complex	Activated	4.373	1.72E-30
BMSCs	IL17A	cytokine	Activated	4.349	1.03E-12
BMSCs	TLR7	transmembrane receptor	Activated	4.344	4.69E-08
BMSCs	IFN ALPHA/BETA (family)	group	Activated	4.316	6.9E-17
BMSCs	G PROTEIN ALPHA I (family)	group	Activated	4.316	2.55E-14
BMSCs	IL27	cytokine	Activated	4.31	0.000000137
BMSCs	CGAS	enzyme	Activated	4.271	3.73E-13
BMSCs	STX18	transporter	Inhibited	-3.9	0.0000238
BMSCs	USP8	peptidase	Inhibited	-3.908	8.68E-08
BMSCs	IRGM	enzyme	Inhibited	-3.911	0.00000176
BMSCs	TOX	transcription regulator	Inhibited	-3.962	9.99E-13
BMSCs	KDM1A	enzyme	Inhibited	-3.996	2.31E-30
BMSCs	Scd2	enzyme	Inhibited	-4.021	1.61E-13
BMSCs	AREG	growth factor	Inhibited	-4.069	4.62E-20
BMSCs	E2F1	transcription regulator	Inhibited	-4.123	2.52E-20
BMSCs	PTGER2	G-protein coupled receptor	Inhibited	-4.148	5.12E-25
BMSCs	MYC	transcription regulator	Inhibited	-4.303	2.18E-17
BMSCs	IRF2BP2	transcription regulator	Inhibited	-4.323	4.64E-13
BMSCs	ERBB2	kinase	Inhibited	-4.332	2.98E-32
BMSCs	BMI1	transcription	Inhibited	-4.398	1.78E-10

		regulator			
BMSCs	EP400	other	Inhibited	-4.423	2.02E-14
BMSCs	CD3E	transmembrane receptor	Inhibited	-4.506	1.88E-12
BMSCs	NFAT5	transcription regulator	Inhibited	-4.533	1.17E-18
BMSCs	F3	transmembrane receptor	Inhibited	-4.533	4.29E-14
BMSCs	PCLAF	other	Inhibited	-4.698	3.66E-22
BMSCs	PNPT1	enzyme	Inhibited	-4.772	3.22E-16
BMSCs	TRIM24	transcription regulator	Inhibited	-4.781	4.36E-11
BMSCs	RNASEH2B	other	Inhibited	-4.886	1.54E-11
BMSCs	E2F (family)	group	Inhibited	-4.973	1.53E-29
BMSCs	TREX1	enzyme	Inhibited	-5.026	2.22E-21
BMSCs	STAT6	transcription regulator	Inhibited	-5.083	8.08E-34
BMSCs	SOCS1	other	Inhibited	-5.122	5.87E-13
BMSCs	FOXM1	transcription regulator	Inhibited	-5.249	1.56E-25
BMSCs	TTC39A-AS1	other	Inhibited	-5.251	1.3E-17
BMSCs	TBX2	transcription regulator	Inhibited	-5.787	6.73E-26
BMSCs	TBX3	transcription regulator	Inhibited	-6.115	1E-48
BMSCs	CKAP2L	other	Inhibited	-6.164	9.81E-36
BMSCs	RABL6	other	Inhibited	-6.164	2.59E-30
BMSCs	TFEB	transcription regulator	Inhibited	-6.294	3.91E-22
BMSCs	CITED2	transcription regulator	Inhibited	-6.68	1.86E-23
BMSCs	Eldr	other	Inhibited	-7.04	5.62E-55
BMSCs	PPARD	ligand-dependent nuclear receptor	Inhibited	-7.7	7.37E-49

References

1. Sanborn, M.A., Wang, X., Gao, S., Dai, Y. & Rehman, J. Unveiling the cell-type-specific landscape of cellular senescence through single-cell transcriptomics using SenePy. *Nat Commun* **16**, 1884 (2025).
2. Saul, D.*, et al.* A new gene set identifies senescent cells and predicts senescence-associated pathways across tissues. *Nat Commun* **13**, 4827 (2022).
